# *Citrobacter rodentium* infection impairs dopamine metabolism and exacerbates the pathology of Parkinson’s disease in mice

**DOI:** 10.1186/s12974-024-03145-0

**Published:** 2024-06-07

**Authors:** Yongtao He, Jiayin Zhao, Yuanyuan Ma, Xin Yan, Yufei Duan, Xiaoshuang Zhang, Hongtian Dong, Rong Fang, Yunhe Zhang, Qing Li, Ping Yang, Mei Yu, Jian Fei, Fang Huang

**Affiliations:** 1 Department of Translational Neuroscience, Jing’an District Centre Hospital of Shanghai, State Key Laboratory of Medical Neurobiology and MOE Frontiers Center for Brain Science, Institutes of Brain Science, Fudan University, 138 Yixueyuan Road, Shanghai, 200032 China; 2 Shanghai Engineering Research Center for Model Organisms, SMOC, Shanghai, 201203 China; 3 School of Life Science and Technology, Tongji University, 1239 Siping Road, Shanghai, 200092 China

**Keywords:** Parkinson’s disease, *Citrobacter rodentium* infection, Inflammatory bowel disease, Neuroinflammation, Microbiota disturbances, Neurotransmitter metabolism, TLR4 signaling pathway

## Abstract

Parkinson’s disease (PD) is a prevalent neurodegenerative disorder with indistinct etiology and ill-defined pathophysiology. Intestinal inflammation involved in the pathogenesis of PD, but the underlying mechanism is not fully understood. *Citrobacter rodentium* (C.R) is a gram-negative bacterium that can be used to induce human inflammatory bowel disease in mice. Here, we investigated whether the proinflammatory effects caused by C.R infection initiate PD-like injury and/or exacerbate PD pathology and extensively studied the underlying mechanism. Mice were gavaged once with C.R and monitored for several pathological features at 9 days post infection. The results showed that C.R delivery in mice induced IBD-like symptoms, including significant weight loss, increased fecal water content, an impaired intestinal barrier, intestinal hyperpermeability and inflammation, and intestinal microbiota disturbances. Notably, C.R infection modified dopamine (DA) metabolism in the brains of both male and female mice. Subsequently, a single high dose of MPTP or normal saline was administered at 6 days post infection. At 3 days after MPTP administration, the feces were collected for 16 S rRNA analysis, and PD-like phenotypes and mechanisms were systemically analyzed. Compared with C.R or MPTP injection alone, the injection of C.R and MPTP combined worsened behavioral performance. Moreover, such combination triggered more severe dopaminergic degeneration and glial cell overactivation in the nigrostriatal pathway of mice. Mechanistically, the combination of C.R and MPTP increased the expression of TLR4 and NF-κB p65 in the colon and striatum and upregulated proinflammatory cytokine expression. Therefore, C.R infection-induced intestinal inflammation can impair dopamine metabolism and exacerbate PD pathological processes.

**Supplementary Information:**

The online version contains supplementary material available at 10.1186/s12974-024-03145-0.

## Introduction

Parkinson’s disease is a progressive irreversible neurodegenerative disorder characterized by dopaminergic neuronal death in the substantia nigra (SN) and the aggregation of α-synuclein (α-syn) in surviving neurons. PD affects more than 10 million people worldwide and there is currently no effective cure for this disease [1]. PD patients present with a range of classic movement disorders, including bradykinesia, rigidity, resting tremors, and postural instability [2]. However, some nonmotor manifestations involving gastrointestinal (GI) dysfunctions such as constipation, dysphagia and nausea often precede motor symptoms in PD patients [3, 4].

The pathogenesis of PD is obscure, but current evidence strongly supports that intestinal inflammation is a cause [5]. As demonstrated by a study in humans, the expression of inflammatory cytokines in colonic biopsy and fecal samples from PD patients was greater than that in samples from healthy individuals [6, 7]. Moreover, epidemiological studies have shown that the incidence of idiopathic PD is approximately 50% lower in chronic users of nonsteroidal anti-inflammatory drugs or cyclo-oxygenase inhibitors than in age-matched individual with normal use of these drugs [8]. Inflammatory bowel disease (IBD), a common type of enteritis, has been confirmed to be a potential risk factor for PD development [9]. For example, epidemiologic studies have reported a significant association between Crohn’s disease and the risk of developing PD [10, 11]. Genome-wide association studies have demonstrated that there are several overlapping of gene loci between PD and IBD [12]. In addition, one study showed that the treatment of IBD with anti-TNF agents reduced PD incidence rates by more than 78% [10]. However, related experiments are rare in animal studies. Only one report at present shows that dextran sulfate sodium (DSS)-evoked ulcerative colitis (UC) can exacerbate lipopolysaccharide-induced damage to the nigral dopaminergic system [13]. Hence, more evidence is needed to verify the association between intestinal inflammation and PD and reveal the detailed underlying mechanisms involved.

C.R is a specific murine pathogenic bacterium that mainly colonizes the colon, resulting in transient colitis [14, 15]. Multiple strains of adult mice, including outbred mice (Swiss Webster) and inbred mice (C57BL/6, C3H and FVB), are susceptible to C.R infection, which induces attaching and effacing lesions [14, 16, 17]. Proinflammatory states of C.R infection are clearly observed [16]. However, the colonic mucosal inflammation induced by C.R infection is more moderate than that induced in other colitis models, such as chemical induced models. A previous report showed more moderate myeloperoxidase scores in C.R*-*infected mice than in DSS-challenged mice [18], suggesting that C.R infected colitis models could better mimic human intestinal diseases. Moreover, in *Pink1* deficient mice, infection with C.R triggers PD-like symptoms by changing the microbial composition [19, 20]. The other detrimental effects of microbial disorders caused by C.R infection on the PD process, however, have not been fully elucidated.

In this study, we utilized C.R gavage to establish an intestinal inflammation model and confirmed the induction of IBD-like symptoms through analysis of several pathological features. Additionally, we investigated whether C.R-induced intestinal inflammation impairs dopamine metabolism and exacerbates the loss of dopamine neurons in an MPTP-based model of PD and elucidated the underlying mechanism.

## Materials and methods

### Animals

Male and female C57BL/6J mice aged 10–12 weeks were obtained from Shanghai Model Organisms Center, INC. The mice were raised (12 h light/dark cycle) under standard conditions (temperature 22 ± 2 °C, humidity 50–60%) with free access to food and water. All animal experimental procedures were performed following the Institutional Animal Care and Use Committee of Fudan University, Shanghai Medical College (IACUC Animal Project Number: 20190221-141). All operations were performed under general anesthesia, and all efforts were made to minimize suffering.

### Bacterial culture and infection of mice

C.R (DBS770 strain, chloramphenicol-resistant) was purchased from American Type Culture Collection (ATCC) and cultured according to previously described methods [21]. Briefly, a single colony was picked after culturing C.R on chloramphenicol MacConkey agar plates (LA8200, Solarbio life sciences, China) for 24 h at 37 ℃. Then, bacteria were shaken overnight in Luria-Bertani (LB) broth supplemented with chloramphenicol (50 µg/mL) at 200 × g at 37 ℃ and collected the bacteria by centrifugation at 3000 × g for 10 min. Pelleted bacteria were washed and resuspended in 0.01 M sterile PBS. The concentration of bacteria was assessed by measuring absorbance at an optical density (OD) of 600 nm and serially diluted and plated each inoculation culture to confirm the colony-forming units (CFUs).

Mice were fasted and only given water for 8–12 h before oral gavage with 4 × 10^8^ CFU in volume of 200 µL. The mice were allowed ad libitum access to food after and weighted daily after gavage. To monitor bacterial burden, fresh feces were collected from each mouse at 2-day intervals, weighed, and dissociated in 0.01 M sterile PBS at a ratio of 0.1 g stool per 1 mL PBS. The supernatant was collected after centrifugation at 400 × g for 3 min at 4 °C, and plated on chloramphenicol MacConkey agar plates after serial dilution. Incubated the plates at 37 °C overnight to allow colonies to growth, and counted colonies. Final counts were presented as CFU/g and plotted on a log scale in Prism.

To further explore the effector components of C.R, the bacteria were cultured to OD600 around 0.4–0.5 and centrifuged at 3000 × g for 10 min. The supernatant was collected and then passed through 0.22 μm polyether-sulfone filters to remove the residual bacterial cells. Finally, the bacterial pellet was resuspended in sterile PBS and then sterilized at 121 ℃ for 5 min, to get inactivated bacteria concentration (4 × 10^8^ CFU/ 200 µL).

### C.R identification by PCR

Total DNA was extracted from *Citrobacter rodentium* and *Escherichia coli* (ATCC25922) via boiling at 100 ℃ in ddH_2_O. The primers: 5’-GCTTAATGGAATTGGTCAGGCC-3’ (*EspF*-F) and 5’-GCGAGAGGGAGTTAATGACG-3’ (*EspF*-R) against *Citrobacter rodentium EspF* gene were applied.

### Establishment of Parkinson’s disease mouse model

1-Methyl-4-phenyl-1,2,3,6-tetrahydropyridine (MPTP) (M0896, Sigma-Aldrich, USA) was dissolved in 0.9% saline to a final concentration 5 mg/mL. Considering that the combination of C.R and an acute regimen of MPTP (four consecutive 10–20 mg/kg injections at 2 h intervals) sharply increased mortality, a single injection of high -dose MPTP (40 mg/kg) was given by intraperitoneal administration, and the control animals identically were received the same volume of 0.9% saline. 3 days after MPTP administration, mice were sacrificed after performing behavioral tests (treatment time in the study is depicted in Table S2).

### Behavioral tests

All behavioral tests were performed on Day 3 after MPTP treatment.

#### Pole test

The mice stood on a rough-surfaced wooden pole (50 cm in height and 1 cm in diameter) placed in the home cage, and some buffer material was placed at the bottom to prevent falling injury. A training trial was performed before the experiment. The turning time and time to descend to the bottom of the pole were recorded for each mouse in 3 trials with an interval of 1 h. The final results represented the average values of 3 trials.

#### Rotarod test

Mice were placed on a rotarod apparatus, and then the rod revolved at a speed of 32 rpm for up to 300 s. The time for each mouse to fall off the rod was recorded. Each mouse performed the test for 3 trials with an interval of 1 h. The final data represented the average values of 3 trials.

#### Wire-hanging test

The wire hanging test was carried out according to a previously described method with slight modifications [22]. In brief, the mice were suspended on their forelimbs on a horizontal wire (1.6 mm in diameter, 50 cm long, 30 cm high between two poles equipped with a platform). The animals were trained for 2 days with 3 trials per day before the experiment. The baseline score was 10 points, and falling from the wire resulted in a one-point deduction, whereas climbing to the platform resulted in the addition of one point. This test was carried out in 3 trials, and the final scores were an average of scores in the 3 trials.

#### Open field test (OFT)

The animals were placed in an open-field apparatus consisting of a white cube opaque chamber (40 × 40 × 40 cm) and allowed to freely move for 5 min. All activities were recorded by a computer system. The total moving distance, mean velocity, frequency of movements and time spent in the central zone were analyzed to evaluate motor function and emotion.

#### Rearing test

The mice were placed into a transparent cylinder (25 cm in diameter, 60 cm high) for 3 min, and the number of rearing were recorded blindly by an experimenter.

### Fecal water and food intake measurements

At 9 days after C.R infection, the animals were placed individually in a clean and transparent plastic cage for 1 h. The fresh feces were collected into a tube, and the wet weight was measured. The dry weight was determined after 24 h of drying at 85 °C. The water content percentage was calculated as the wet weight minus the dry weight divided by the wet weight. In addition, food intake was calculated over a 24-h period before sacrifice.

### Measurement of intestinal transmission distance and colon length

The gastrointestinal function was assessed by measurement of intestinal transmission distance [23]. In brief, at 9 days after C.R infection, animals fasted for 8 h followed by oral administration (10 µL/g) of the suspension containing 5% activated carbon (934,674, Sigma-Aldrich, USA) and 5% Gum Arabic (G9752, Sigma-Aldrich, USA). 30 min later, the mice were sacrificed and the small intestine was isolated. Then, the total length of the small intestine, and the distance from the pylorus to the black marker were measured, which represented intestinal transmission distance.

Meanwhile, the colon length between the end of the cecum and the anus was measured.

### Intestinal permeability assay

At 9 days after C.R infection, the intestinal barrier integrity was measured by permeability assay using fluorescein isothiocyanate-dextran (FITC)-dextran (FD4, Sigma-Aldrich, USA). In brief, mice fasted for 4 h and then they were orally given FD4 at 0.6 mg/g body weight. After another 4 h, mice were euthanized and blood was collected from the orbital cavity. The sample was kept at room temperature for 2 h without light and the serum was collected after centrifugation at 3000 × g for 10 min. The fluorescence intensity was detected by a fluorescence spectrophotometer (excitation: 485 nm and emission: 528 nm) (PerkinElmer, USA) and FD4 concentration in the serum was further calculated according to a standard curve established in advance.

### Enzyme-linked immunosorbent assay (ELISA)

The ELISA kits used to detect the level of mouse IL-6 (EMC004.96, Neobioscience, China) in serum and calprotectin (KBR-hlk7175, Keborui, China) in feces at 9 days after C.R infection. The operations were conducted according to the manufacturer’s instructions. IL-6 and calprotectin concentrations were expressed in pg/mL and ng/g, respectively.

### Western blotting

Mouse striatal and colonic proteins were extracted at 3 days after C.R plus MPTP administration via lysing in RIPA buffer (P0013B, Thermo Fisher Scientific, USA) with protease and phosphatase inhibitor (78,425, Thermo Fisher Scientific, USA). The total protein concentration of the supernatant was measured by a BCA Kit (23,227, Thermo Fisher Scientific, USA). After the supernatant was denatured by boiling for 5 min in 1 × loading buffer, equal amounts of protein samples were subjected to 7.5–12.5% SDS-polyacrylamide gel electrophoresis and transferred to polyvinylidene difluoride membranes. The membranes were blocked with 5% non-fat milk in 0.1% TBST for 1 h at room temperature before incubation with primary antibodies overnight at 4 °C. After washing with 0.1% TBST, the membranes were incubated with an IRDye® 800CW goat anti-mouse or anti-rabbit IgG (1:20000; 92,632,210, 92,632,211, LI-COR, USA) for 1 h at room temperature. The blots were visualized by an infrared imaging system (Li-Cor, USA) and the relative level of protein was quantified using Image J software (NIH, Bethesda, MD). The primary antibodies used in the study including: mouse anti-TH antibody (1:1000; T2928, Sigma-Aldrich, USA), rabbit anti-GFAP antibody (1:1000; 16825-1-AP, Proteintech, USA), rabbit anti-Iba1 antibody (1:1000; ab178846, Abcam, USA) and rabbit anti-COX2 antibody (1:1000; ab179800, Abcam, USA), mouse anti-β-actin antibody (1:2000; sc-47,778, Santa Cruz, USA), rabbit anti-DAT antibody (1:1000; ab184451, Abcam, USA), rabbit anti-MAOA antibody (1:1000; ab12675, Abcam, USA), rabbit anti-MAOB antibody (1:1000; 12602-1-AP, Proteintech, USA), mouse anti-TLR4 antibody (1:500; sc-293,072, Santa Cruz, USA), mouse anti-NF-κB p65 antibody (1:500; ab32536, Abcam, USA), mouse anti-NF-κB p65 antibody (1:500; ab32536, Abcam, USA), rabbit anti-phospho-NF-κB p65 (Ser536) antibody (1:1000; 93H1, Cell Signaling Technology, USA).

### Immunofluorescence and immunochemical staining

At 3 days after MPTP administration, the animals were deeply anesthetized and perfused through the ascending aorta with 0.9% saline infusion. The brain and colon were dissected and immediately fixed in a 4% paraformaldehyde (PFA) fixative solution for 24 h. Next, the tissues were dehydrated (in 20% and 30% sucrose overnight at 4 °C, respectively), embedded in OCT and sectioned (30 μm thickness) on a frozen microtome (Leica, Germany). The sections were permeabilized and blocked with 0.2% Triton X-100 and 10% normal goat serum in PBS at 37 °C for 1 h, and subsequently incubated with primary antibodies as follows: mouse anti-TH antibody (1:1000; T2928, Sigma-Aldrich, USA), rabbit anti-GFAP antibody (1:1000; 16825-1-AP, Proteintech, USA), rabbit anti-Iba1 antibody (1:1000; ab178846, Abcam, USA), rabbit anti-C3 antibody (1:1000, ab200999, Abcam, USA), rat anti-CD16/32 antibody (1:300; 553,142, BD Pharmingen, USA), rabbit anti-ZO-1 antibody (1:200; sc-33,725, Santa Cruz, USA), rabbit anti-Occludin antibody (1:200; sc-133,256, Santa Cruz, USA), rabbit anti-Claudin 1 antibody (1:200; sc-166,338, Santa Cruz, USA), mouse anti-TLR4 antibody (1:200; sc-293,072, Santa Cruz, USA) overnight at 4 °C. The sections were incubated with Alexa Fluor 488-conjugated, 594 conjugated-, or Cy3-conjugated secondary antibodies (1:1000; A-11,008, A-11,012, A10522, Invitrogen, USA) for 2 h before image capture. DAPI solution was performed to stain nuclei.

For immunochemical staining, the sections were incubated with 0.3% H_2_O_2_ to reduce endogenous peroxidase activity, subjected to antigen retrieval with sodium citrate solution (pH = 6.0), and blocked with PBS containing 0.2% Triton X-100 and 10% normal goat serum at 37 °C for 45 min. Then the sections were incubated overnight at 4 °C with primary antibodies, including mouse anti-TH (1:1000; T2928, Sigma-Aldrich, USA), rabbit anti-GFAP (1:1000; 16825-1-AP, Proteintech, USA), and rabbit anti-Iba1 antibodies (1:1000; ab178846, Abcam, USA). The following day, the sections were incubated with a biotin-conjugated goat anti-rabbit or goat anti-mouse IgG secondary antibody (1:200; PK-6101, PK-6102, Vector laboratories, USA), and staining was performed using an ABC (avidin-biotin complex) system. The reaction was detected with a DAB substrate kit (1:200; SK-4100, Vector laboratories, USA). Images were acquired by a microscope (Olympus DP73, Japan). Optical density of striatal TH-positive fibers was quantitatively evaluated using Image-Pro Plus 6.0 software (Media Cybernetics, USA) and the density and morphology of striatal microglia was determined using Image J software (NIH, Bethesda, MD).

### Stereological counting of TH immunoreactivity

To calculate the number of TH-positive cells in the SN at 3 days after C.R and MPTP combined treatment, stereological cell counting was performed as previously described [24]. The total number of TH neurons in the SN was estimated using the Stereo Investigator system (Micro Brightfield, USA) which combined with an Olympus microscope (Tokyo, Japan). Briefly, a total of 6 Sect. (30 μm thickness/section) at a 5-section interval between Bregma − 2.80 and − 3.65 mm were chosen. A specified outline of SN was delimited at 4 × objectives, and the cells were sampled at 40 × objectives. Stereological counting was analyzed by an experimenter blinded to the experimental groups. The final data are presented as a total number of TH neurons.

### RNA isolation and quantitative real-time-PCR analysis

Total RNA was extracted from striatum and colon samples with TRIzol isolation reagent (DP424, Tiangen, China) according to the manufacturer’s instructions, and RNA was reverse-transcribed to cDNA using a high-capacity cDNA reverse transcription kit (KR116, Tiangen, China). The target mRNA amount in the samples was detected using SYBR-Green (CW3008M, CWBIO, China) and the relative mRNA expression levels of various genes were determined by quantitative real-time polymerase chain reaction (qPCR) on QuantStudio 3 Instrument (Applied Biosystems, USA) with the following 3-step thermal cycling protocol: denaturation was performed at 95 ℃ for 30 s (step1), followed by annealing at 60 ℃ for 10 s (step2) and extension subsequently occurred at 72 ℃ for of 30 s (step3). The 3-step process was repeated for a total of 40 cycles. Data collection occurs at the 72 ℃ stage for 30 s for each of the cycles across all wells. Normalized to the mRNA expression level of β-actin, the mRNA expression of other genes was calculated using the 2^−ΔΔCt^ method. The paired primers used for amplification are listed in Table  S1.

### Measurement of neurotransmitters and SCFAs by high-performance liquid chromatography (HPLC)

At 9, 15, 21 and 30 days after C.R infection, the isolated striatum was homogenized in 0.4 M HClO_4_ and then centrifuged at 12,000 × g, at 4 °C for 10 min. The supernatants were collected to determine the concentrations of neurotransmitters, and 20 µL of the sample was injected into an Acclaim™ 120 C18 column (Thermo Fisher Scientific, USA). The concentrations of DA, 5-HT and their metabolites, including dihydroxyphenylacetic acid (DOPAC), homovanillic acid (HVA) and 5-hydroxyindoleacetic acid (5-HIAA), were quantified by HPLC via chromatography (ESA, USA) with a 5014B electrochemical detector.

The fresh feces were subjected to ether extraction to obtain SCFAs. Briefly, approximately 0.3 g of fresh feces was collected and thoroughly suspended in ddH2O. After centrifugation at 12,000 × g for 10 min, the supernatant was collected, and 100 µL of concentrated hydrochloric acid was added. Then, 5 mL of ether was added, and the mixture was allowed to react for 20 min at room temperature. The resulting organic phase solution was collected and mixed with 0.5 mL of 1 M NaOH solution. After thorough mixing, the mixture was extracted for an additional 20 min. The aqueous phase solution was then collected by centrifuging the mixture at 3500 × g for 10 min. Subsequently, the aqueous phase was filtered through a 0.22 μm organic filter membrane and the concentrations of acetic acid, propionic acid, and butyric acid were determined using HPLC. The chromatographic conditions involved mobile phase A (0.01% phosphoric acid aqueous solution) and mobile phase B (methanol), with linear gradient elution. The detection wavelength was set at 210 nm, the column temperature was 30 °C, and the flow rate was 1 mL/min. The results are presented in units of µg/mg of tissue.

### Gut microbe 16 S rRNA sequencing and data analysis

Fresh fecal pellets were collected at 9 days after C.R infection or 3 days after MPTP administration, and immediately stored at -80 °C for further analysis. Total genomic DNA was extracted from fecal samples with a QIAamp Fast DNA Stool Mini Kit. The DNA concentration was determined with an Equalbit dsDNA HS Assay Kit. The V3-V4 regions of the microbial 16 S rRNA were amplified using a panel of proprietary primers aimed at relatively conserved regions designed by GENEWIZ (Suzhou, China). All the quantified amplicons were pooled together at equal concentrations for Illumina MiSeq sequencing (Illumina, San Diego, CA, USA). Experiments including DNA extraction, quality assessment, library construction, and high-throughput sequencing were performed by GENEWIZ.

The raw sequencing data were filtered, and sequences longer than 200 bp were retained. Only high-quality sequences without chimeras were used for analysis. VSEARCH clustering (1.9.6) was used to cluster the high-quality sequences with 97% similarity to obtain operational taxonomic units (OTUs). Then representative sequences of OTUs were annotated by comparison to a database (Silva 132).

α-Diversity indices (ACE index and Shannon index) were calculated by QIIME (V1.9.1) based on the OTU results. β-Diversity analysis was performed to investigate the structural variation in microbial communities across samples using UniFrac distance metrics, and the results were visualized via principal coordinate analysis (PCoA). Differences in the UniFrac distances among groups were determined by analysis of similarities (ANOSIM). LEfSe was performed to detect differentially abundant taxa at the phylum, family, and genus levels across groups using the default parameters.

### Histological analysis

After the mice was euthanized at 9 days after C.R infection, the colon was removed and washed once with ice-cold PBS. The terminal 0.5-cm piece of the colon was immersed in 4% formaldehyde for 24 h and embedded in paraffin, after which and 5-micron sections were cut. The sections were deparaffinized and dehydrated with xylene and decreasing concentrations of ethanol (100%, 90%, 80%, and 70% ethanol) for subsequent Hematoxylin and Eosin (H&E) staining. Images were taken by a microscope (Olympus DP73, Japan). The histological score was blindly assessed by 2 experimenters. The scoring criteria were based on published literature [25]. Briefly, histological changes in the intestines, including inflammation and epithelial injury, were graded. Inflammatory infiltration was evaluated based on the severity and extent of lymphocyte and neutrophil infiltration. Epithelial injury was assessed according to the degree of tissue damage, loss of mucosal glands, and the presence of ulceration or erosion. Histopathological changes for each parameter were scored using a scale from 0 to 3, with 0 indicating no changes, 1 indicating mild changes, 2 indicating moderate changes, and 3 indicating severe changes. The area involved was rated as follows: 0 = no involvement, 1 ≤ 25% of the section, 2 ≤ 50%, 3 ≤ 75%, and 4 ≤ 100%.

### Statistical analyses

Statistical analyses were performed with Prism 7 (GraphPad software, USA). All the data were represented as the mean ± S.E.M. and assessed for normal distribution by the Shapiro-Wilk test. Nonparametric test was used when the data did not fit normal distribution. For experiments involving only two groups, an unpaired *t* test was used for comparison. Depending on the number of independent variables, one-way or two-way ANOVA followed by LSD multiple comparison tests were used. The correlations between different experiments utilized the Pearson correlation analysis. Statistically significant differences were defined as *p* < 0.05.

## Results

### C.R infection causes gut microbiota alterations

C.R is a pathogen restricted to mice, and it shares 67% of its genes with human enteric pathogens [26]. C.R colonies were identified as pink colonies with narrow white trim on MacConkey agar plates (Fig. S1 A). Specific primers against the C.R *EspF* gene were applied for identification [27] (Fig. S1 B). The growth curve of C.R was established, and the bacterial concentration (CFU/mL) in the culture was calculated from the OD600 values (Fig. S1C-D).

**Fig. 1:**
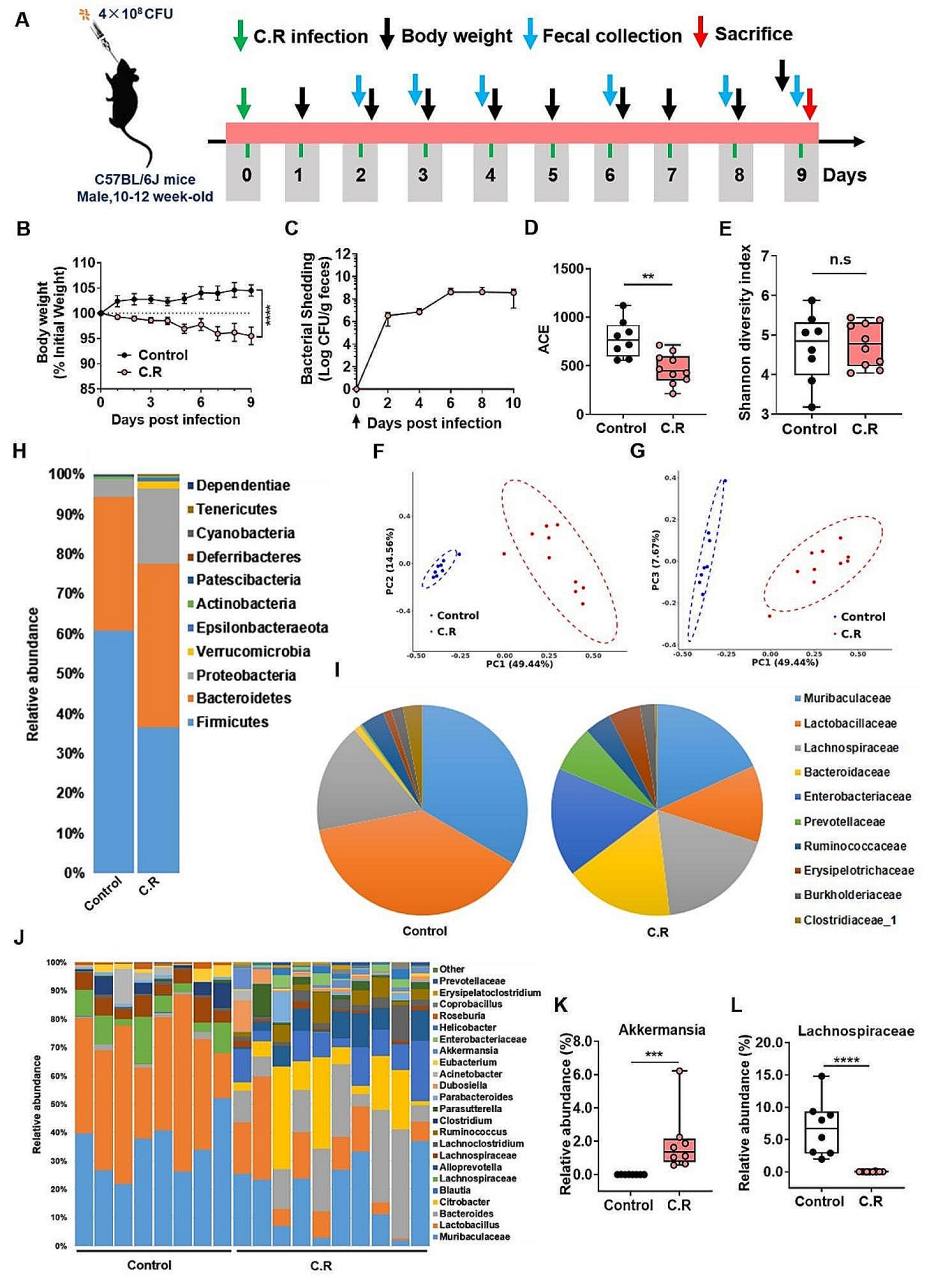
C.R infection model establishment and analysis of the gut microbiome. **(A)** The scheme of C.R oral inoculation in the study. **(B)** The change in body weight relative to the initial weight of the mice was analyzed at the indicated time points after infection with C.R. *n* = 10–26. **(C)** Bacterial shedding in feces was monitored at 2-day intervals after infection with C.R. *n* = 10. **(D-E)** The analysis of α-Diversity based on 16 S rRNA sequencing predicted gut microbiota richness according to the ACE index **(D)** and diversity according to the Shannon index **(E)**. **(F-G)** PCoA plots based on weighted UniFrac metrics of the gut microbiota where samples of mice from different groups are highlighted with different colors. PC1 and PC2 explained 49.44% and 14.56%, respectively, of the variance. PC1 and PC3 explain 49.44% and 7.67%, respectively, of the variance. The position and distance of the data points indicated the degree of similarity in terms of both the presence and relative abundance of the bacterial taxonomies. **(H)** Percentage stacking chart based on the Bray-Curtis distance analysis of the differences in the relative abundance of gut microbiota at the phylum level between the groups. **(I)** Differences in the relative abundance of gut microbiota at the family level between the groups. **(J)** Differences in the relative abundances of gut microbiota at the genus level between the groups. **(K-L)** Relative abundances of 2 significantly altered bacterial genera: *Akkermansia*** (K)**, and *Lachnospiraceae*** (L)**. *n* = 8–10. **p < 0.01, ***p < 0.001, ****p < 0.0001

Mice were orally administered with 4 × 10^8^ CFU of C.R after fasting, and different parameters were recorded and analyzed according to the experimental scheme to confirm the effectiveness of C.R infection **(**Fig. 1A**)**. A significant decrease in body weight was observed after C.R infection. At 9 days post infection (d.p.i.), the body weight of the mice was approximately 5% less than the initial weight **(**Fig. 1B**)**. The fecal load of C.R was measured at 2-day intervals. The amount of C.R reached approximately 10^9^ CFU/g in feces at 6 d.p.i and remained relatively constant until the end of monitoring **(**Fig. 1C**)**. The fecal water content was monitored at 3-day intervals. Compared with uninfected mice with approximately 58% fecal water content, C.R-infected mice exhibited an increase in the percentage of water in the feces (63% at 3 d.p.i., 73% at 6 d.p.i., and 68% at 9 d.p.i.) and a maximum at 6 d.p.i. (C.R vs. control: *p* < 0.0001) (Fig.  S2A). The food consumption of the animals was monitored at 9 d.p.i., and a notable decrease was observed in C.R-infected mice compared to the control mice (C.R vs. control: *p* = 0.0004) (Fig.  S2B). The length of small intestinal transit was evaluated and there was a significant reduction compared to that in the control group (C.R vs. control: *p* = 0.0375) (Fig.  S2C). In contrast, the spleen index of C.R-infected mice was greater than that of control group mice (C.R vs. control: *p* = 0.0017) (Fig.  S2D), suggesting an increased immune response. Collectively, these data confirmed the effectiveness of C.R infection.

To investigate the effect of C.R challenge on the gut microbiota composition, we characterized changes in the gut microbiota composition in fecal samples from mice infected with C.R or vehicle solution (control group) at 9 d.p.i. according to high-throughput sequencing of the V3-V4 region of the 16 S rRNA genes. A significant reduction in α-Diversity was observed in the C.R-infected mice compared to the control mice based on the ACE index, an estimate of microbial community richness (C.R vs. control: *p* = 0.0016) **(**Fig. 1D**)**. However, there were no differences between the groups in terms of the Shannon index, which is representative of microbial diversity **(**Fig. 1E**)**. The extent of similarity of the gut microbial communities between the control group and the C.R group was measured using PCoA based on the weighted UniFrac distance and the Bray-Curtis dissimilarity to identify possible differences between the bacterial components in the gut microbiota. PCoA revealed that the gut microbiota of the C.R-infected mice was distinct from that of control mice **(**Fig. 1F-G**)**. These findings indicated that the composition of the gut microbiota in C.R-infected mice was significantly different from that in control mice.

To further identify the critical bacteria affected by C.R colonization, we compared the relative abundances of microbes at various taxon levels between the groups. At the phylum level **(**Fig. 1H**)**, the abundance of *Firmicutes* was significantly decreased in the C.R group compared to the control group (C.R vs. control: *p* < 0.0001) (Fig. S3A). In contrast, other phyla including *Bacteroidetes*, *Verrucomicrobia*, *Proteobacteria* and *Epsilonbacteraeota*, had higher abundances in the C.R group than the control group (C.R vs. control: *p* = 0.0420, *p* = 0.0219, *p* = 0.0048, *p* = 0.0087, respectively) (Fig. S3B-E). Moreover, the ratio of *Bacteroidetes* to *Firmicutes* was significantly higher in the C.R group than in the control group (C.R vs. control: *p* = 0.0067) **(**Fig. 1F**)**. At the family level, as illustrated in the pie chart **(**Fig. 1I**)**, the abundances of *Bacteroidaceae*, *Enterobacteriaceae*, *Erysipelotrichaceae*, *Lachnospiraceae*, and *Prevotellaceae* after the C.R challenge was increased compared to those in the control group, while the levels of *Muribaculaceae*, *Lactobacillaceae* and *Clostridiaceae* were reduced in the C.R group. As shown in Fig. 1J, overall significant differences in genera were detected between the groups. We further selected representative genera and utilized boxplots to display their detailed information **(**Fig. 1K-L and Fig. S3G-N). Compared to those in the control group, the relative abundances of *Akkermansia* (*p* = 0.0106) **(**Fig. 1K**)**, *Citrobacter* (*p* = 0.0034) (Fig. S3G), *Ruminococcus* (*p* = 0.0035) (Fig. S3H), *Helicobacter* (*p* = 0.0087) (Fig. S3I), *Coprobacillus* (*p* = 0.0155) (Fig. S3G) and *Parasutterella* (*p* = 0.0117) (Fig. S3K) were significantly increased in the C.R group, whereas the abundances of the genera *Lachnospiraceae* (*p* = 0.0001) **(**Fig. 1L**)**, *Oscillibacter* (*p* = 0.0219) (Fig. S3L), *Clostridium* (*p* = 0.0145) (Fig. S3M), and *Roseburia* (*p* = 0.0002) (Fig. S3N) were notably decreased in the C.R group. Collectively, these results indicated a significantly altered microbiota profile in C.R-infected mice.

### C.R colonization damages the intestinal barrier and induces intestinal inflammation

**Fig. 2:**
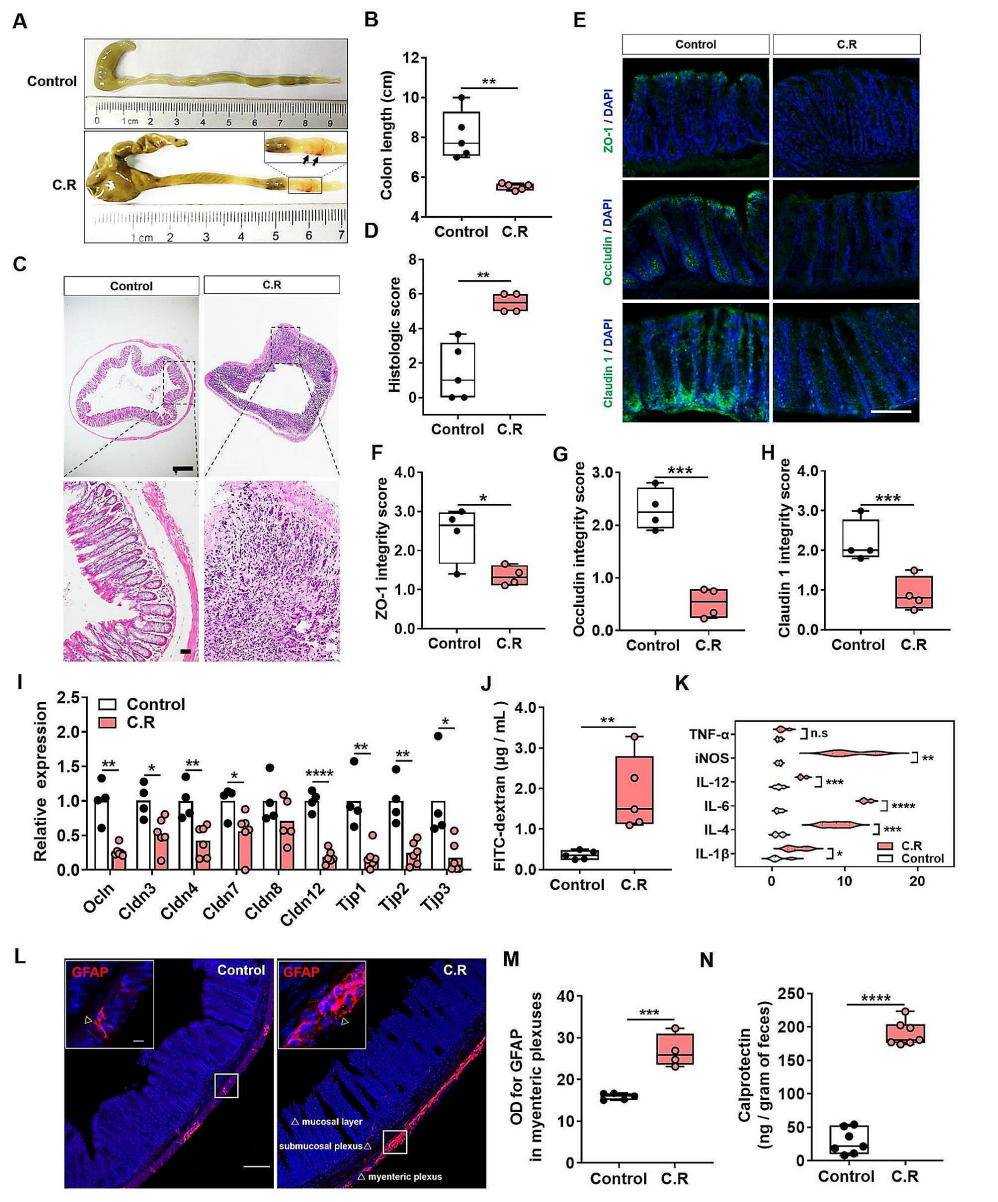
The intestinal barrier damage and intestinal inflammation induced by C.R infection. **(A)** The colonic morphology of the control and C.R-infected mice. The arrows indicate lesion sites in the distal colon. **(B)** The colon length. *n* = 5. **(C)** H&E staining of colons. Scale bar, 200 μm (top) and 20 μm (bottom). **(D)** Histopathological scoring of colonic tissue. *n* = 4–5. **(E)** Representative immunofluorescence images of the tight junction proteins ZO-1, Occludin, and Claudin 1 in colon tissue. Scale bar, 100 μm. **(F-H)** An arbitrary scale of 0–3 (0 = no expression, 3 = continuous normal expression of the barrier) was used to assess barrier integrity. **(F)** Integrity scoring data for ZO-1. **(G)** Integrity scoring data for Occludin. **(H)** Integrity scoring data for Claudin 1. *n* = 4. **(I)** The expression of nine tight junction proteins in colonic tissue was analyzed by qPCR. *n* = 4–6. **(J)** The serum concentrations of FD4. *n* = 5. **(K)** The levels of six inflammatory factors in colonic tissue were analyzed by qPCR. *n* = 3–5. **(L)** Representative images of GFAP^+^ cells in the myenteric plexuses. Normal: scale bar,100 μm; Magnification: scale bar, 10 μm. The triangle arrow indicates GFAP^+^ cells in the myenteric plexuses. **(M)** Optical density analysis of GFAP expression in the myenteric plexuses. *n* = 4–5. **(N)** The level of calprotectin in feces was analyzed by ELISA. *n* = 7. *p < 0.05, **p < 0.01, ***p < 0.001, ****p < 0.0001

C.R is located mainly in the distal part of the colon and cecum during the steady-state phase [28]. To explore the destructive effect of C.R colonization on the intestine, the morphology and pathology of the 0.5-cm terminal colon were evaluated at 9 days after infection. Morphologically, there was an evident lesion indicated by the swelling of the distal colon of C.R-infected mice, while colons of the control mice were normal and filled with feces **(**Fig. 2A**)**. Simultaneously, the C.R-infected mice exhibited a significant decrease in colon length (C.R vs. control: *p* < 0.0001) **(**Fig. 2B**)**. To better define the basis of these differences, the terminal colon was examined by H&E staining. The colon mucosa of the control mice displayed an intact epithelium and no overt hyperplasia, while C.R-infected mice showed significant damage, as demonstrated by widespread epithelial cell sloughing, crypt hyperplasia, submucosal edema and extensive polymorphonuclear cell infiltration **(**Fig. 2C**)**. Correspondingly, when the pathology was scored, the score of tissue damage in the colon of C.R-infected mice was significantly higher than that of the untreated mice (C.R vs. control: *p* = 0.0024) **(**Fig. 2D**)**. Goblet cell depletion was also detected in the colon tissue (Fig. S4).

At the molecular level, intestinal barrier dysfunction in the colon was evaluated via immunofluorescence staining for the tight junction proteins zonula occludens-1 (ZO-1), Occludin, and Claudin-1 **(**Fig. 2E**)**. The pattern showed continuous, robust expression of ZO-1 at the epithelial lining of the control mice while the C.R-infected mice exhibited a disrupted, discontinuous structure of ZO-1 **(**Fig. 2E**)**. Semiquantitative analysis of the staining using an integrity scoring scale demonstrated a notable decrease after C.R infection (C.R vs. control: *p* = 0.0296) **(**Fig. 2F**)**. Microscopic analysis revealed a stronger Occludin staining in the epithelial lining of the control group **(**Fig. 2E**)**. The integrity scores for Occludin expression were significantly lower after C.R infection (C.R vs. control: *p* = 0.0003) **(**Fig. 2G**)**. Microscopic visualization revealed that Claudin-1 was clearly expressed in the colonic epithelial lining of the control group, and Claudin-1 expression was significantly impacted by C.R infection **(**Fig. 2E**)**. The analysis also revealed significantly lower expression of Claudin-1 in C.R-infected mice than in control mice (C.R vs. control: *p* = 0.0093) **(**Fig. 2H**)**. Consistently, the mRNA expression of multiple tight junction-related genes in the colon was significantly decreased in the C.R-infected group (C.R vs. control: *Ocln*, *p* = 0.0004; *Cldn3*, *p* = 0.0146; *Cldn4*, *p* = 0.0062; *Cldn7*, *p* = 0.0356; *Cldn8*, *p* = 0.2302; *Cldn12*, *p* < 0.0001; *Tjp1*, *p* = 0.0022; *Tjp2*, *p* = 0.0014; *Tjp3*, *p* = 0.0176) **(**Fig. 2I**)**. In addition, an intestinal permeability assay was conducted in vivo through oral delivery of 4 KDa FITC-dextran (FD4), and a greater increase in FD4 in the serum represented elevated gut permeability. The results showed that there was a remarkable increase in FD4 intensity after C.R challenge (C.R vs. control: *p* = 0.0065) **(**Fig. 2J**)**. All these data suggested that C.R colonization caused intestinal barrier impairment.

Immunofluorescence staining revealed a different pattern of GFAP^**+**^ enteric glial reactivity in the myenteric plexuses of the colon tissue at 9 days post infection **(**Fig. 2L**)**. GFAP intensity in the lamina propria of C.R-infected mice was significantly higher than that in the control mice (C.R vs. control: *p* = 0.0005) **(**Fig. 2M**)**. In addition, the colon of the C.R-infected mice showed a considerably increased mRNA expression of proinflammatory factors, including *IL-1β* (*p* = 0.0237), *IL-6* (*p* < 0.0001), *IL-12* (*p* = 0.0004), *iNOS* (*p* = 0.0011), and the anti-inflammatory factor *IL-4* (*p* = 0.0005), but decreased mRNA expression of the *TNF-α* (*p* = 0.1587) compared to the colon of control mice **(**Fig. 2K**)**. Fecal calprotectin has been used as an indicator of intestinal inflammation [29]. ELISA data showed that the level of calprotectin in the fecal pellets of C.R-infected mice was significantly higher than that in the control mice (C.R vs. control: *p* < 0.0001) **(**Fig. 2N**)**. Taken together, these data demonstrated that C.R colonization induced intestinal inflammation.

### C.R infection regulates the metabolism of short-chain fatty acids in the gut and neurotransmitters in the brain

**Fig. 3:**
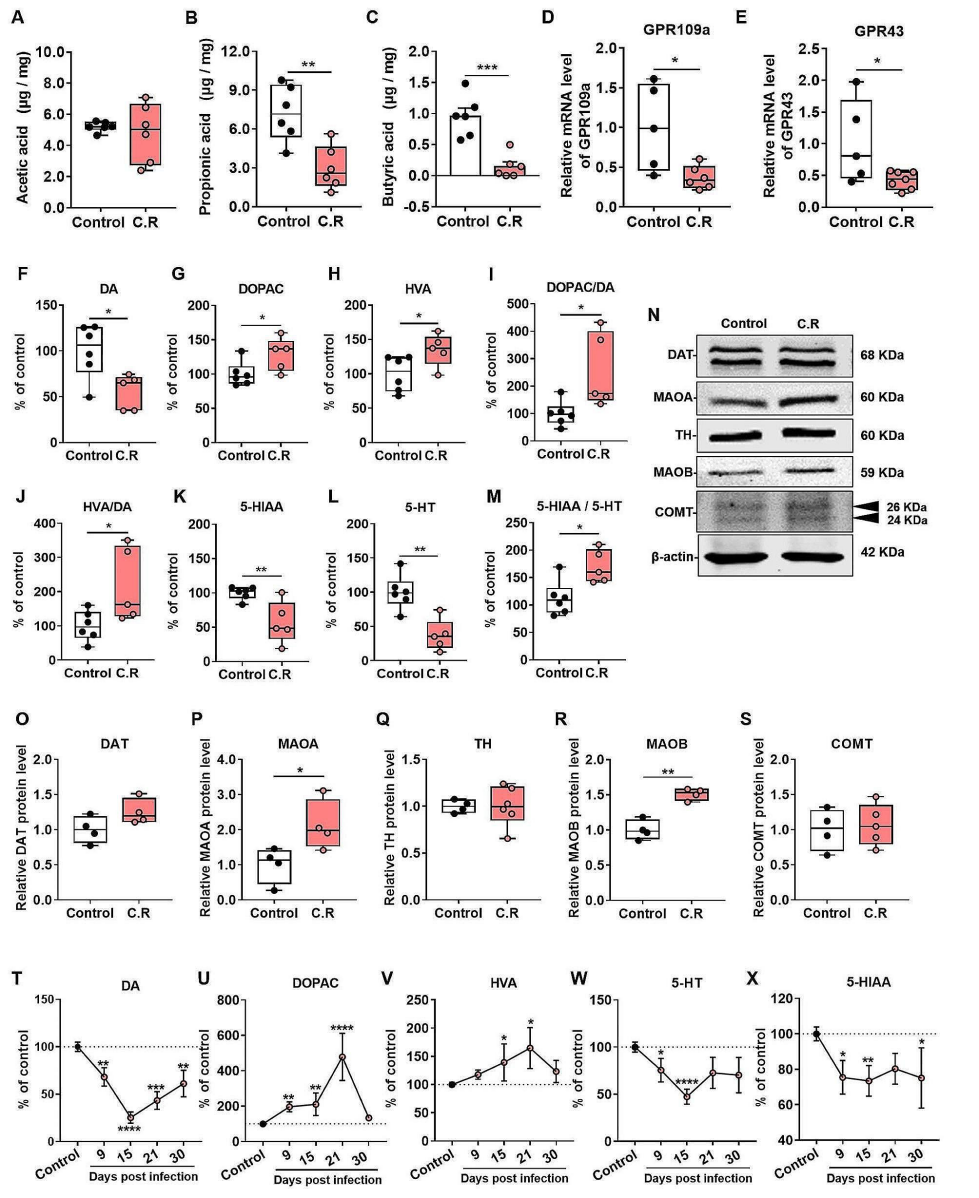
C.R infection caused metabolic abnormalities in SCFAs in the gut and in neurotransmitters in the striatum. **(A-C)** The levels of acetic acid **(A)**, propionic acid **(B)**, and butyric acid **(C)** in feces at 9 days post infection. *n* = 6. **(D, E)** The mRNA levels of *GPR109a***(D)** and *GPR43***(E)** in colonic tissue. *n* = 5–7. **(F-M)** The metabolism of DA and 5-HT. DA **(F)**, DOPAC **(G)**, HVA **(H)**, DOPAC/DA **(I)**, HVA/DA **(J)**, 5-HIAA **(K)**, 5-HT**(L)**, and 5-HIAA/5-HT **(M)**. *n* = 5–6. **(N)** Western blotting showing striatal levels of the DAT, MAOA, TH, MAOB and COMT proteins. β-actin served as the loading control. **(O-S)** Quantification of the relative expression levels of the DAT **(O)**, MAOA **(P)**, TH **(Q)**, MAOB **(R)** and COMT **(S)** proteins. *n* = 3–6. **(T-X)** The metabolism of DA and 5-HT at 9, 15, 21, and 30 days post infection. DA **(T)**, DOPAC **(U)**, HVA **(V)**, 5-HT**(W)**, 5-HIAA **(X)**. *n* = 3–10. *p < 0.05, **p < 0.01, ***p < 0.001, ****p < 0.0001

Mounting evidence has demonstrated that SCFAs are involved in the regulation of intestinal inflammation [30]. To explore the potential mechanisms of action of C.R challenge in gut inflammation, the fresh fecal samples were collected for the evaluation of SCFAs by HPLC. The results showed that compared to that in the control mice, the concentration of propionic acid (C.R vs. control: *p* = 0.0034) **(**Fig. 3B**)** and butyric acid (C.R vs. control: *p* = 0.0004) **(**Fig. 3C**)** exhibited a notable decrease while the level of acetic acid **(**Fig. 3A**)** remained unchanged in the C.R-infected mice. Moreover, the SCFA receptors GPR109a and GPR43 were detected in the colon. The data showed that there was lower expression of *GPR109a* and *GPR43* in the C.R group than in the control group (C.R vs. control: *p* = 0.0218 and *p* = 0.0396, respectively) **(**Fig. 3D-E**)**. In brief, these data suggested that C.R infection affected SCFA metabolism in the gut.

Gut microbiota alterations is linked to the disturbance of neurotransmitter metabolism [31]. To verify the effect after C.R infection, the metabolism of tyrosine (Tyr) and tryptophan (Trp) in the striatum was assessed at 9 days after C.R colonization. Surprisingly, the DA level was decreased (C.R vs. control: *p* = 0.0183) **(**Fig. 3F**)**, while the levels of DOPAC (C.R vs. control: *p* = 0.0496) **(**Fig. 3G**)**, HVA (C.R vs. control: *p* = 0.0445) **(**Fig. 3H**)** were markedly increased in the Tyr metabolism pathway.

To further explore the underlying mechanism, the step limiting enzyme tyrosine hydroxylase (TH), MAOA, MAOB, catechol-O-methyltransferase (COMT) and dopamine transporter (DAT) were investigated (Fig. 3N**)**. We found that the expression of TH, DAT and COMT did not change (C.R vs. control) **(**Fig. 3Q, O and S), while MAOA (C.R vs. control: *p* = 0.0142) **(**Fig. 3P**)** and MAOB (C.R vs. control: *p* = 0.0142) **(**Fig. 3R**)** expression was markedly increased. In the Trp metabolism pathway, the levels of the metabolites 5-HT and 5-HIAA in C.R-infected mice were markedly lower than those in the control mice (C.R vs. control: *p* = 0.0021 and *p* = 0.0094, respectively) **(**Fig. 3L and K). Additionally, the ratios of DOPAC/DA (C.R vs. control: *p* = 0.0271) **(**Fig. 3I**)**, and HVA/DA (C.R vs. control: *p* = 0.0368) **(**Fig. 3J**)**, and 5-HIAA/5-HT (C.R vs. control: *p* = 0.0131) **(**Fig. 3M**)** were obviously increased, indicating increased turnover of DA and 5-HT in C.R infection. In addition, similar trends in Tyr and Trp metabolism were also observed in the female mice (Fig. S5).

To further explore whether there was a continuous and long-term effect on neurotransmitter metabolism in the striatum, the animals were sacrificed at 9, 15, 21 and 30 days after C.R challenge. The results showed that there was a remarkable decrease in DA in the C.R-infected group in comparison with the control group, with the lowest level being observed at 15 d.p.i. **(**Fig. 3T**)**. In contrary, the levels of DOPAC and HVA increased during C.R infection compared to the control group, with the peaks levels of both metabolites appearing at 21 d.p.i. (Fig. 3U and V). At the end of monitoring, the DOPAC and HVA levels returned to baseline **(**Fig. 3U and V). The levels of 5-HT and its metabolite 5-HIAA in the C.R group were lower than those in the control group. After C.R colonization for 15 days, 5-HT levels were gradually returned to normal **(**Fig. 3W**)**, while 5-HIAA levels showed a continuous decline **(**Fig. 3X**)**.

To further validate the effective components of C.R gavage, the Luria Broth (LB), supernatant and precipitate of C.R culture medium were given by gavage according to the manufacturer’s instructions (Fig. S6A). The levels of neurotransmitters and metabolites (DA, DOPAC, HVA, 5-HT and 5-HIAA) in the striatum were evaluated by HPLC after 12 days of continuous treatment. Compared with those in LB-treated mice, the five substances remained unchanged in mice treated with the supernatant or the precipitate (Fig. S6B). Moreover, there was no change in the expression of the TH and MAOB proteins after supernatant and precipitate treatment compared with that in the LB-treated group (Fig. S6C-E). Taken together, these results indicated that only living C.R delivery disturbs neurotransmitter metabolism.

### C.R in combination with MPTP challenge causes motor deficits

**Fig. 4:**
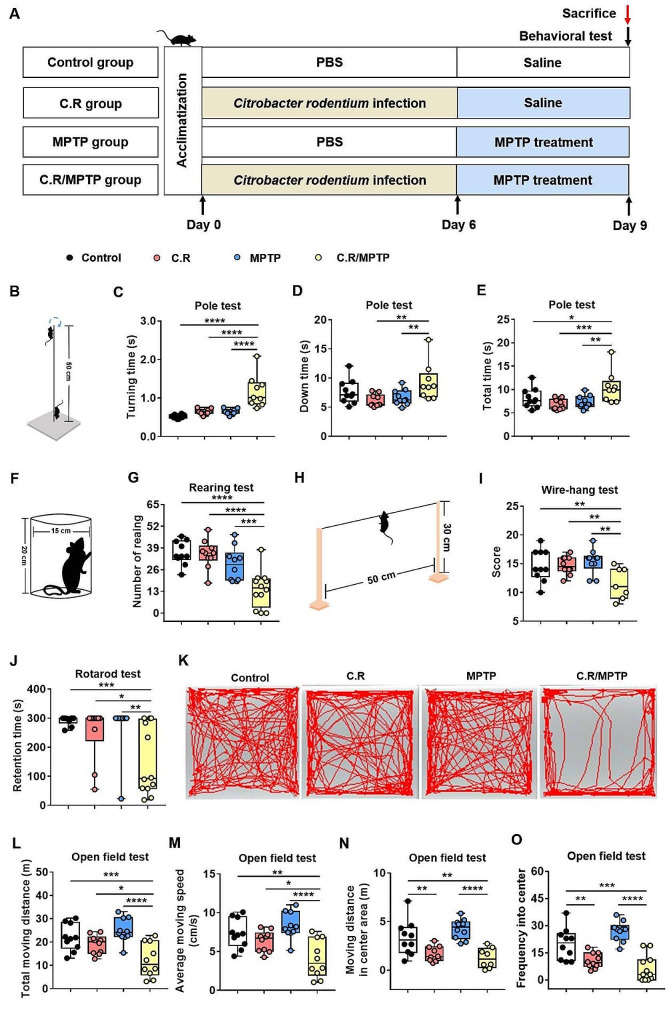
The combination of C.R and MPTP caused movement impairments in PD mice. **(A)** The experimental design in the current study. **(B-E)** Behavioral changes were assessed in the pole test **(B)**, including the time to turning **(C)**, time to reach the bottom **(D)** and the total time **(E)**. **(F-G)** Behavioral changes were assessed in the rearing test **(F)** and number of rearing in a 3-minute period **(G)**. **(H-I)** Behavioral changes were assessed via the wire-hanging test **(H)** and the total score over 3 min **(I)**. **(J)** Behavioral changes were assessed in the rotarod test and the retention time on the rod at speed of 32 rpm was recorded. **(K-O)** Behavioral changes were assessed in the open field test; representative traces **(K)**, total moving distance **(L)**, average moving speed **(M)**, moving distance in the center area **(N)**, and frequency in the center **(O)** are shown. *n* = 7–12. *p < 0.05, **p < 0.01, ***p < 0.001, ****p < 0.0001

As mentioned in the literature, imbalances in neurotransmitter metabolism and the gut microbiota are causal factors of PD pathogeny [32]. To evaluate the effects of intestinal disorders on PD pathogenesis, we established an infection mouse model by orally giving mice C.R once. At 6 days post inoculation, the C.R-challenged mice were administrated 40 mg/kg MPTP or vehicle **(**Fig. 4A**)**. At 9 d.p.i. (3 days after MPTP administration), various behavioral tests, including the pole test, the rotarod test, the wire-hanging test and the rearing test, were performed to assess motor function. Overall, there were no significant behavioral changes in these mice, regardless of treatment with C.R or MPTP alone compared to the control group **(**Fig. 4B-J**)**. However, the mice administered C.R and MPTP combined exhibited significant motor disorders, including delayed initiation of movement, as indicated by a longer turning time (C.R/MPTP vs. control: *p* < 0.0001) **(**Fig. 4C**)**, more time spent climbing down the pole (C.R/MPTP vs. control: *p* = 0.0077) **(**Fig. 4D**)** and the whole climbing process in the pole rest (C.R/MPTP vs. control: *p* = 0.0024) **(**Fig. 4E**)**, the motor deficit characterized by less standing time in the rearing test (C.R/MPTP vs. control: *p* = 0.0006) **(**Fig. 4G**)**, poorer muscle force manifested as a lower score in the wire-hanging test (C.R/MPTP vs. control: *p* = 0.0020) **(**Fig. 4I**)**, and decreased retention time on the rod (C.R/MPTP vs. control: *p* = 0.0034) **(**Fig. 4J**)** in the rotarod test.

In addition, the open field test (OFT) was also performed to evaluate locomotion and emotion. The data showed that there were no marked changes in distances traveled or the speed of the mice treated with C.R alone or MPTP alone compared with those of the control mice **(**Fig. 4K-M**)** in the OFT. C.R in combination with MPTP challenge prominently caused less movement (C.R/MPTP vs. control: *p* < 0.0001) **(**Fig. 4L**)** and bradykinesia (C.R/MPTP vs. control: *p* < 0.0001) **(**Fig. 4M**)**. C.R alone or C.R and MPTP combined challenge led to shorter distances in the central area (C.R vs. control: *p* = 0.0045, C.R/MPTP vs. control: *p* < 0.0001) **(**Fig. 4N**)** and a lower frequency in the central area (C.R vs. control: *p* = 0.0036, C.R/MPTP vs. control: *p* < 0.0001) **(**Fig. 4O**)** than vehicle. The OFT results suggested that C.R infection also participated in emotional regulation. Taken together, these data suggested that C.R and MPTP induced motor impairments and slight anxiety-like behavior.

### C.R infection in combination with MPTP exacerbates the loss of TH-positive dopaminergic neurons and TH protein expression in the brain

**Fig. 5:**
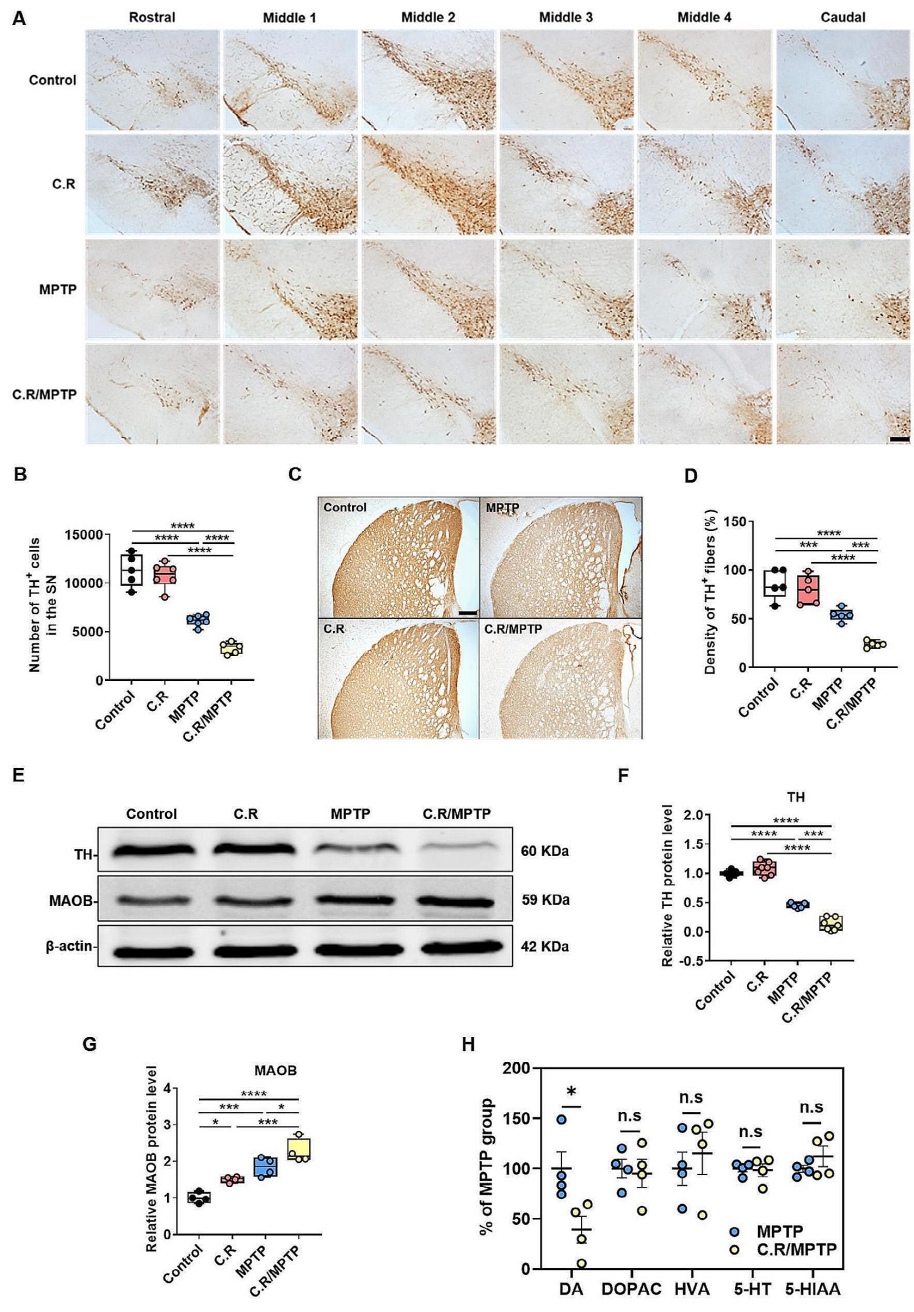
C.R infection aggravated MPTP-induced nigrostriatal system lesions. **(A)** Immunohistochemical staining showing TH^**+**^ cells in the SN. **(B)** Results of stereological counting of TH^**+**^ cells in the SN. Scale bar, 100 μm. *n* = 5–6. **(C)** Immunohistochemical staining showing striatal TH^**+**^ nerve fibers. Scale bar, 200 μm. **(D)** Densitometric analysis of the relative optical density of the staining. *n* = 5. **(E)** Western blotting showing the striatal levels of the TH and MAOB proteins. β-Actin served as the loading control. **(F, G)** Quantification of the relative protein expression levels of TH and MAOB. TH: *n* = 5–7; MAOB: *n* = 3–4. **(H)** The levels of DA, DOPAC, HVA, 5-HT and 5-HIAA in the striatum. *n* = 4. *p < 0.05, **p < 0.01, ***p < 0.001, ****p < 0.0001

Given that dopaminergic neuronal loss in the nigrostriatal system is the main pathological characteristic of PD, we examined whether C.R infection also has a destructive effect on the dopaminergic system in the SN and the striatum. Tyrosine hydroxylase (TH) is an enzyme involved in DA synthesis; therefore, TH^**+**^ cells are dopaminergic neurons. Representative images of immunohistochemical staining of the SN rostral to caudal in the coronal section are shown in Fig. 5A. By stereological counting analysis of the SN, we found that the number of TH^**+**^ cells decreased by nearly 50% after MPTP exposure alone (MPTP vs. control: *p* < 0.0001), while no significant difference was detected in mice with C.R infection alone compared to the control mice **(**Fig. 5B**)**. However, C.R in combination with MPTP significantly exacerbated the loss of dopaminergic neurons in PD mice (C.R/MPTP vs. MPTP: *p* = 0.0006) **(**Fig. 5B**)**. Consistent with this observation, immunostaining and western blotting of the striatum also revealed marked decreases in the TH^**+**^ fiber (MPTP vs. control: *p* = 0.0004) and TH protein (MPTP vs. control: *p* < 0.0001) levels in the MPTP group compared to those in the control group, while there was no change in mice challenged with C.R alone compared to the control mice **(**Fig. 5C-D and E-F, respectively). Nevertheless, there was a more severe decrease in TH^**+**^ fibers and TH protein expression in the C.R/MPTP group compared to the MPTP-challenged group (C.R/MPTP vs. MPTP: TH^**+**^ fibers, *p* = 0.0006; TH protein levels, *p* < 0.0001) **(**Fig. 5C-D and E-F, respectively). MAOB is responsible for the enzymatic degradation of dopamine at axon terminals, often showing a negative correlation with TH expression. There were an elevated protein levels of striatal MAOB in only C.R exposed or only MPTP exposed mice compared to the control mice (C.R vs. control: *p* = 0.0392; MPTP vs. control: *p* = 0.0012) **(**Fig. 5E-G**)**. However, C.R in combination with MPTP significantly increased MAOB expression in comparison with MPTP alone (C.R/MPTP vs. MPTP: *p* = 0.0250) **(**Fig. 5E-G**)**. Moreover, the level of DA in the striatum was significantly decreased in the C.R/MPTP group compared to the MPTP group (C.R/MPTP vs. MPTP: *p* = 0.0300) **(**Fig. 5H**)**, while the levels of the DA metabolites DOPAC and HVA were not different between the groups **(**Fig. 5H**)**. The levels of 5-HT and its metabolite 5-HIAA also did not differ between the MPTP group and the C.R/MPTP group **(**Fig. 5H**)**.Taken together, these data confirm that peripheral C.R infection aggravated the nigrostriatal dopaminergic system lesions.

### Activation of microglia in the SN and the striatum is exacerbated after C.R and MPTP combination treatment

**Fig. 6:**
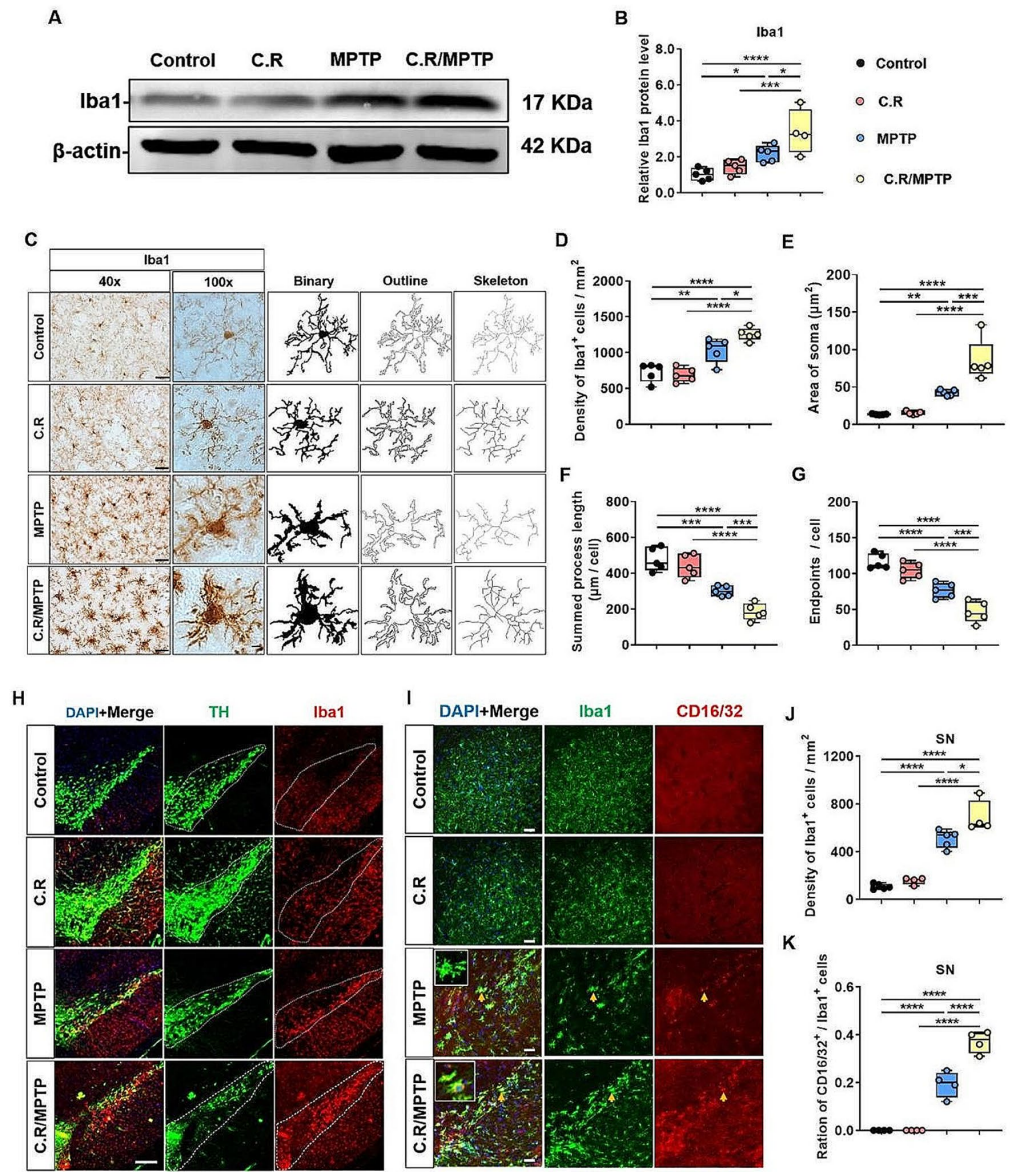
The C.R challenge exacerbated MPTP-induced microglial activation in the striatum and the SN. **(A)** Western blotting showing striatal levels of Iba1 proteins. β-Actin served as the loading control. **(B)** Quantification of the relative protein expression levels of Iba1. *n* = 4–5. **(C)** Immunohistochemical staining showing Iba1^**+**^ microglial cells in the striatum. Scale bar, 40×: 20 μm, 100×: 5 μm. **(D)** The number of Iba1^**+**^ cells. *n* = 5. **(E-G)** Morphological analysis of Iba1^**+**^ cells, the soma area **(E)**, the summed process length **(F)**, and the endpoints of microglia **(G)**. *n* = 5. **(H)** Immunofluorescence staining of TH (green), Iba1 (red) and DAPI (blue) in the SN. Scale bar, 100 μm. **(I)** Immunofluorescence staining of Iba1 (green), CD16/32 (red) and DAPI (blue) in the SN. Scale bar, 200 μm. **(J)** The numbers of Iba1^**+**^ cells in the SN. **(K)** The ratio of the number of CD16/32^**+**^ and Iba1^**+**^ cells to the total number of Iba1^**+**^ cells. *n* = 4–5. *p < 0.05, **p < 0.01, ***p < 0.001, ****p < 0.0001

Iba1 is a marker of microglia. We first used Western blotting analysis to evaluate the expression of Iba1 in the striatum. The results demonstrated that the level of Iba1 protein was greater in the MPTP group than in the control group (MPTP vs. control: *p* = 0.0146) **(**Fig. 6A-B**)**, and the C.R treatment alone did not affect Iba1 expression **(**Fig. 6A-B**)**. In the C.R/MPTP group, the Iba1 protein level was significantly increased compared to that in the MPTP group (C.R/MPTP vs. MPTP: *p* = 0.0164) **(**Fig. 6A-B**)**.

Next, we evaluated the number and morphology of microglia within the striatum via immunohistochemical assays. The results showed that Iba1^**+**^ cells in the control group or the C.R group were characterized by a small number of highly-ramified morphologies **(**Fig. 6C**)**. After MPTP intoxication or C.R and MPTP combined treatment, numerous activated Iba1^**+**^ microglia with stouter cell processes and a round shape were observed **(**Fig. 6C**)**. In terms of quantity, the number of Iba1^**+**^ cells were markedly greater in MPTP-challenged mice than the control mice (MPTP vs. control: *p* = 0.0015) **(**Fig. 6C-D**)**. Conversely, there was no impact on mice challenged with C.R alone **(**Fig. 6C-D**)**. However, in the C.R/MPTP group, the number of Iba1-labeled microglia was significantly higher than that in the MPTP group (C.R/MPTP vs. MPTP: *p* = 0.0135) **(**Fig. 6C-D**)**.

Furthermore, the morphology of the microglia was analyzed. There was no obvious difference between the C.R group and the control group **(**Fig. 6E-G**)**. Compared with those in the control group, the soma of Iba1-labeled microglia in the MPTP-treated group were enlarged (MPTP vs. control: *p* = 0.0046) **(**Fig. 6E**)**, the summed process length (MPTP vs. control: *p* < 0.0001) **(**Fig. 6F**)** and total number of endpoints (MPTP vs. control: *p* < 0.0001) **(**Fig. 6G**)** were decreased. Strikingly, in the C.R/MPTP group, activated microglia exhibited a larger soma area (MPTP vs. control: *p* = 0.0002) **(**Fig. 6E**)**, a shorter summed process length (MPTP vs. control: *p* = 0.0034) **(**Fig. 6F**)** and fewer endpoints of microglia (MPTP vs. control: *p* = 0.0010) than did the MPTP group **(**Fig. 6G**)**.

Similar results were obtained when microglia in the SN were analyzed. In the present study, we performed immunofluorescence staining of the SN region to detect the activation of glial cells, using Iba1 as a marker of microglia **(**Fig. 6H**)**. The number of Iba1^**+**^ cells was significantly greater in the MPTP group than in the control group (MPTP vs. control: *p* < 0.0001) **(**Fig. 6J**)**. No effect on Iba1^**+**^ cells was observed in mice infected with C.R alone **(**Fig. 6J**)**. However, more Iba1^**+**^ cells were found in mice challenged with C.R and MPTP than in mice challenged with MPTP alone (C.R/MPTP vs. MPTP: *p* = 0.0033) **(**Fig. 6J**)**.

To evaluate the classic M1 phenotype of microglia in the SN, the expression of CD16/32, a typical marker of the M1 activation, was analyzed. The data showed that there were no obvious CD16/32-labeled microglia in the SN in the control or C.R-infected mice **(**Fig. 6I and K). There were more CD16/32-labeled cells in the MPTP group than in the control group (MPTP vs. control: *p* < 0.0001) **(**Fig. 6I and K). In the C.R/MPTP group, the number of CD16/32-labeled cells was greater than that in the MPTP group (C.R/MPTP vs. MPTP: *p* < 0.0001) **(**Fig. 6I and K).Collectively, these results illustrated that peripheral C.R infection significantly exacerbated MPTP-induced microglial activation in the nigrostriatal pathway.

### Activation of astrocytes in the SN and striatum is exacerbated after C.R and MPTP combined challenge

**Fig. 7:**
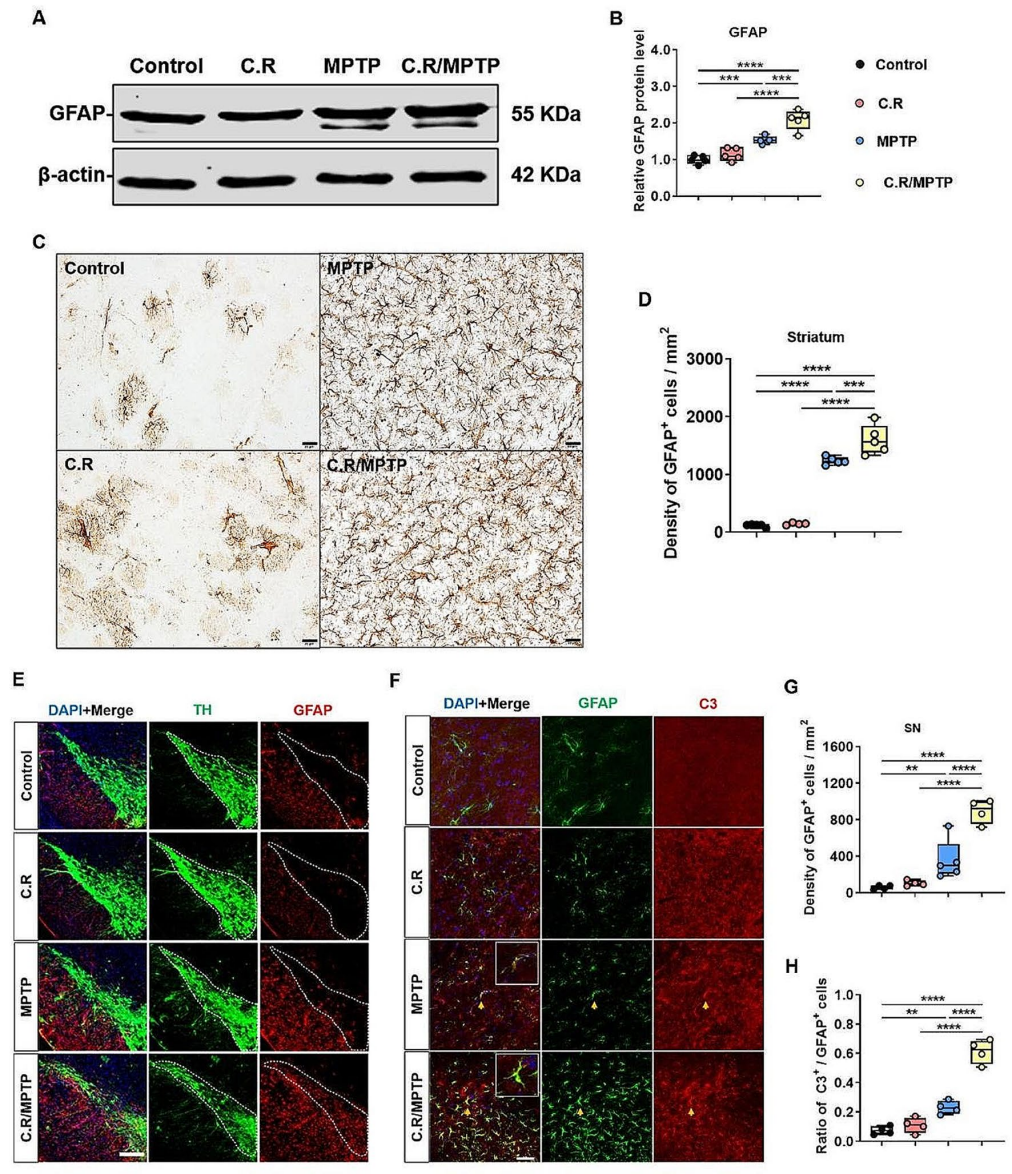
The combination of C.R and MPTP exacerbated neurotoxicity-mediated astrocyte activation in the nigrostriatal pathway at 9 days post infection. **(A)** Western blotting showing striatal levels of GFAP protein. β-Actin served as the loading control. **(B)** Quantification of the relative expression levels of GFAP protein. *n* = 5. **(C)** Immunohistochemical staining showing GFAP in the striatum. Scale bar, 20 μm. **(D)** The number of GFAP^**+**^ cells in the striatum. *n* = 4–5. **(E)** Immunofluorescence staining of TH (green), GFAP (red) and DAPI (blue) in the SN. Scale bar, 200 μm. **(F)** Immunofluorescence staining of GFAP (green), C3 (red) and DAPI (blue) in the SN. Scale bar, 50 μm. **(G)** Number of GFAP^**+**^ astrocytes in the SN. **(H)** The ratio of the number of C3^**+**^ and GFAP^**+**^ cells to the total number of GFAP^**+**^ cells. *n* = 3–5. *p < 0.05, **p < 0.01, ***p < 0.001, ****p < 0.0001

Astrocytes, another important type of glial cell, play critical roles in homeostasis in the brain. We analyzed astrocyte activation by assessing GFAP expression. Western blotting data showed revealed greater levels of GFAP protein in the striata of MPTP-delivered mice than in those of the control mice (MPTP vs. control: *p* = 0.0002) **(**Fig. 7A, B**)**, while C.R alone had no effect on GFAP expression **(**Fig. 7A, B**)**. Strikingly, the protein level of GFAP was dramatically higher in the C.R/MPTP group than the MPTP group (C.R/MPTP vs. MPTP: *p* = 0.0001) **(**Fig. 7A, B**)**. Moreover, the number of GFAP^**+**^ cells in the striatum was increased, consistent with the protein expression data **(**Fig. 7C, D**)**. Specifically, there were more GFAP^**+**^ cells in the C.R/MPTP group than the MPTP group (C.R/MPTP vs. MPTP: *p* = 0.0008) **(**Fig. 7D**)**. In addition, similar results were acquired in the SN by immunofluorescence staining and cell counting **(**Fig. 7E and G).

To evaluate the neurotoxic effects on the A1 phenotype of astrocytes in the SN, we analyzed C3, a marker of A1 activation. There were more C3^**+**^ cells in the MPTP group than in the control group (MPTP vs. control: *p* = 0.0019) **(**Fig. 7F and H). In the C.R/MPTP group, the number of C3-labeled cells was markedly higher than that in the MPTP group (C.R/MPTP vs. MPTP: *p* < 0.0001) **(**Fig. 7F and H). Collectively, these results demonstrated that the C.R challenge dramatically exacerbated neurotoxicity-mediated astrocyte activation in the nigrostriatal pathway.

### C.R administration activates the TLR4 signaling pathway in the colon and the nigrostriatal pathway in the current model system

C.R is gram-negative bacteria that contain LPS on their membrane surface. TLR4 can recognize and respond to LPS, and its downstream pathways dominate the microbiota-gut-brain axis when gastrointestinal infections or systemic bacterial infections occur [33]. To examine the activation status of the TLR4 pathway in the colon and the nigrostriatal pathway, qPCR, western blotting, and immunofluorescence staining were performed.

**Fig. 8:**
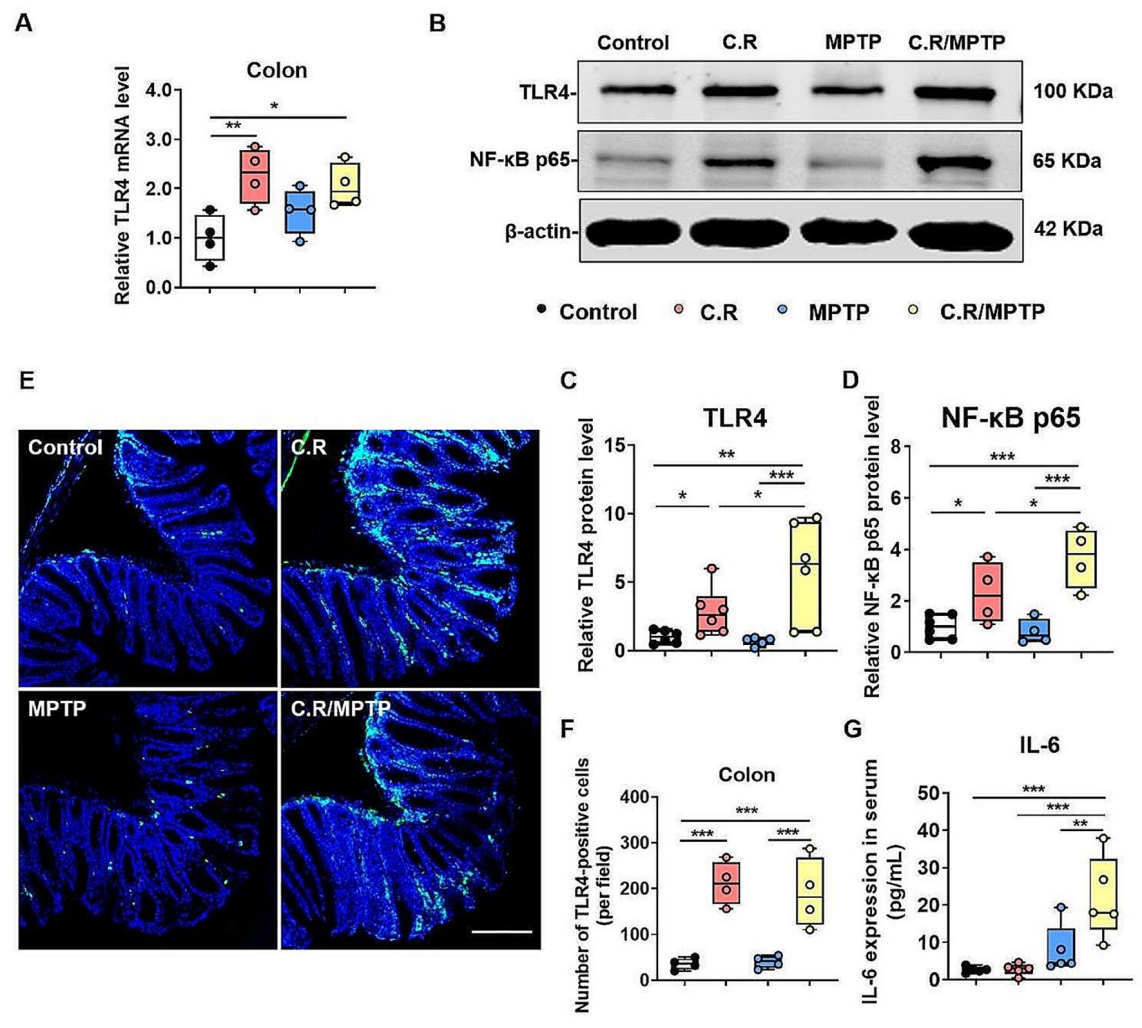
The TLR4 signaling pathway was activated in the colon after the C.R challenge. **(A)** The mRNA level of *TLR4* in the colon. *n* = 4. **(B)** Western blotting showing colonic levels of the TLR4 and NF-κB p65 proteins. β-Actin served as the loading control. **(C-D)** Quantification of the relative protein expression levels of TLR4 and NF-κB p65. *n* = 4–6. **(E)** Immunohistochemical staining showing TLR4 in the colon. Scale bar, 50 μm. **(F)** The number of TLR4^**+**^ cells in the colon in per field. *n* = 4. **(G)** The IL-6 protein level in the serum was analyzed by ELISA. *n* = 5. *p < 0.05, **p < 0.01, ***p < 0.001, ****p < 0.0001

In the colon, the mRNA level of *TLR4* was higher in the C.R (C.R vs. control: *p* = 0.0033) and C.R/MPTP groups (C.R/MPTP vs. control: *p* = 0.0104) **(**Fig. 8A**)** than the control group. As revealed by Western blotting, except for the MPTP group, the C.R and C.R/MPTP groups showed a significantly greater expression of TLR4 and NF-κB p65 protein expression than the control group (C.R vs. control: *p* = 0.0328 and *p* = 0.0333, C.R/MPTP vs. control: *p* = 0.0010 and *p* = 0.0002, respectively) **(**Fig. 8C and D); while the expression of TLR4 and NF-κB p65 in the C.R group was lower than that in the C.R/MPTP group (C.R vs. C.R/MPTP: *p* = 0.0295 and *p* = 0.0371, respectively) **(**Fig. 8C and D). In addition, the immunofluorescence staining results revealed that the numbers of TLR4^**+**^ cells in the colon were notably higher in the C.R and the C.R/MPTP groups than to the control group (C.R vs. control: *p* = 0.0001, C.R/MPTP vs. control: *p* = 0.0005), whereas the number of TLR4^**+**^ cells in the colon of MPTP-injected mice was similar to that in colon of control mice **(**Fig. 8E and F). In addition, we also measured the levels of IL-6 in mouse serum. The ELISA results demonstrated that there was a trend toward an increase in the MPTP group compared to the control group **(**Fig. 8G**)**. However, the C.R/MPTP group showed greater levels of IL-6 than did the MPTP group (C.R/MPTP vs. MPTP: *p* = 0.0035) **(**Fig. 8G**)**.

**Fig. 9:**
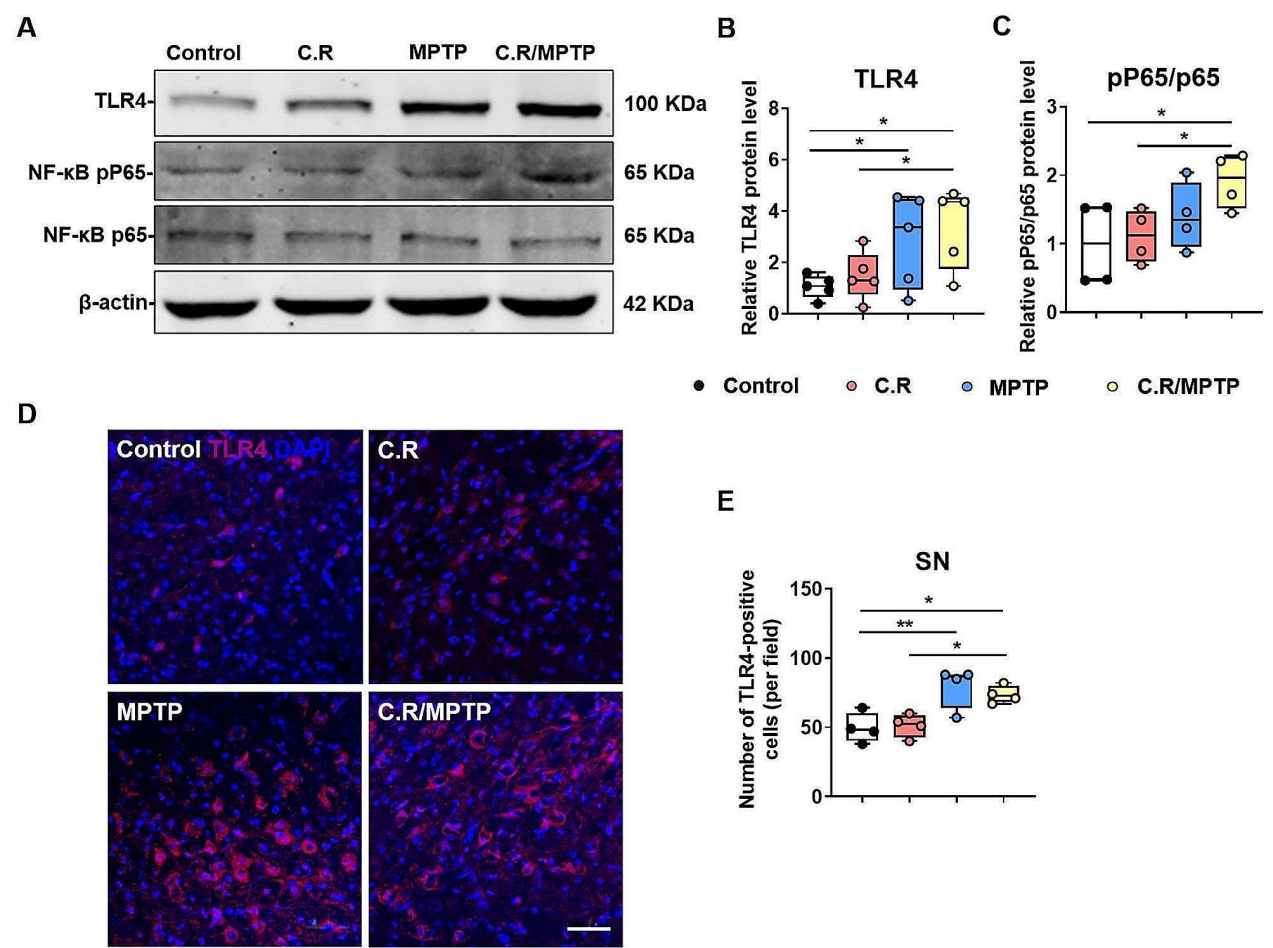
The TLR4-NF-κB signaling pathway was activated in the nigrostriatal pathway in our model system. **(A)** Western blotting showing striatal levels of TLR4, NF-κB p65, and NF-κB pp65 proteins. β-Actin served as the loading control. **(B-C)** Quantification of the relative expression levels of TLR4 and the ratio of NF-κB pp65/p65 proteins. *n* = 5. **(D)** Immunohistochemical staining showing TLR4 in the SN. Scale bar, 20 μm. **(E)** The number of TLR4^**+**^ cells in the SN in per field. *n* = 4. *p < 0.05, **p < 0.01

Similarly, we measured TLR4 expression in the nigrostriatal pathway. In the striatum, the levels of TLR4 (C.R/MPTP vs. control: *p* = 0.0125) and the ratio of NF-κB pp65/p65 (C.R/MPTP vs. control: *p* = 0.0191) were higher in the C.R/MPTP group than the control group **(**Fig. 9A-C**)**. The similar trends in TLR4 expression in the SN were confirmed by immunofluorescence staining (MPTP vs. control: *p* = 0.0018, C.R/MPTP vs. C.R: *p* = 0.0121, C.R/MPTP vs. control: *p* = 0.0078) **(**Fig. 9D-E**)**. All the evidence supported that combined C.R and MPTP challenge activated the TLR4-NF-κB signaling pathway.

### C.R and MPTP combined treatment causes or exacerbates neuroinflammation and alters the gut microbiota composition

**Fig. 10:**
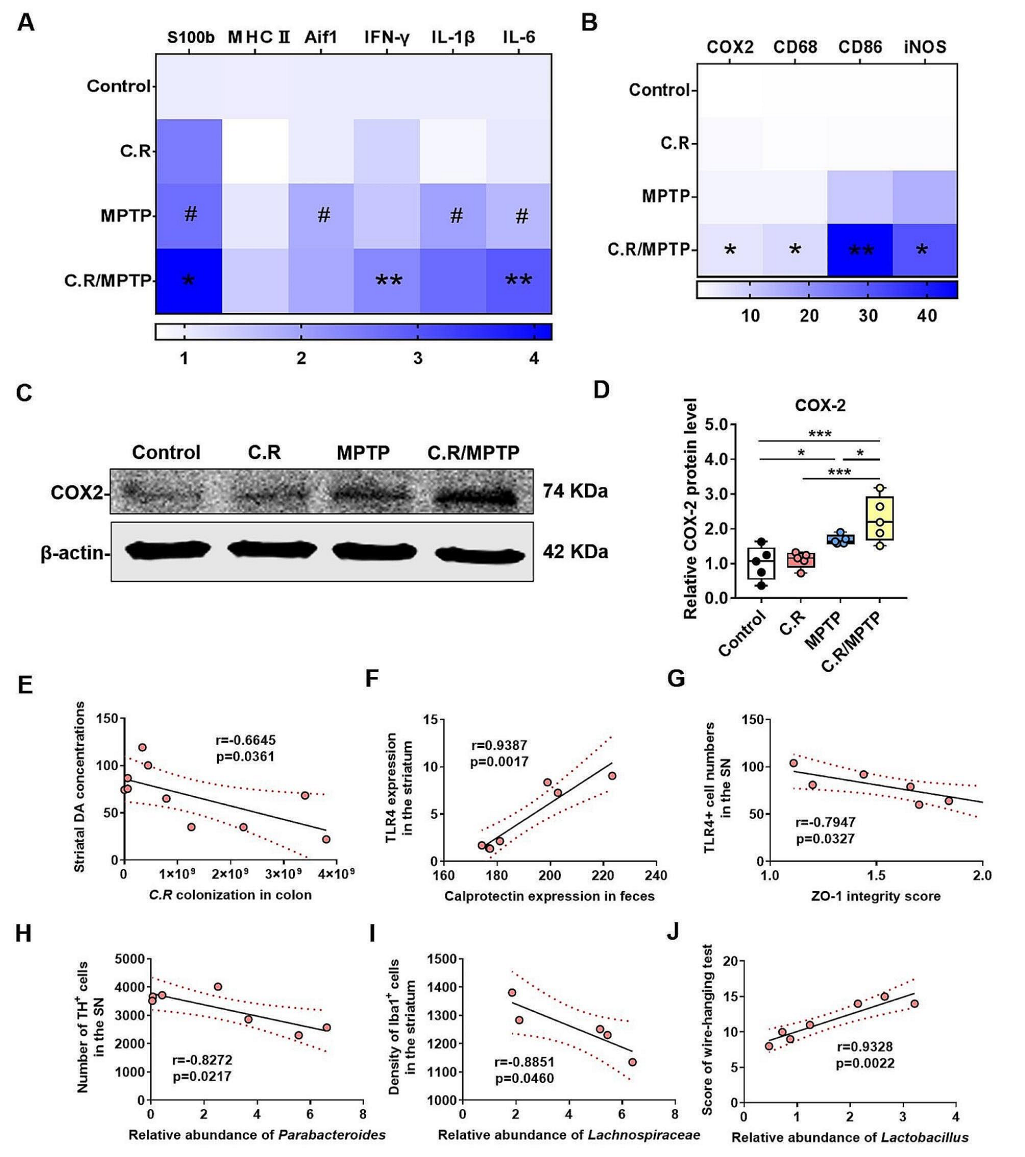
Effect of C.R infection or C.R plus MPTP administration on PD pathology. **(A-B)** The mRNA levels of inflammatory factors in the striatum were analyzed. *n* = 4. ^#^*p* < 0.05, the MPTP group versus the control group; *p < 0.05, **p < 0.01, ***p < 0.001, the MPTP group versus the C.R/MPTP group. **(C)** Western blotting showing striatal levels of COX2. β-Actin served as the loading control. **(D)** Quantification of the relative expression levels of COX2. *n* = 4–5, *p < 0.05, **p < 0.01, ***p < 0.001. **(E)** C.R content in the colon were negatively correlated with the levels of the striatal DA. **(F)** The levels of calprotectin in the feces were positively correlated with TLR4 protein expression in the striatum detected by western blotting. **(G)** The ZO-1 expression in the colon was inversely correlated with TLR4^+^ cell numbers in the SN region. **(H)** The relative abundance of *Parabacteroides* was negatively correlated with TH^+^ neuron numbers in the SN region. **(I)** The relative abundance of *Lachnospiraceae* was inversely correlated with TH^+^ neuron numbers in the SN region. **(J)** The performances of the wire-hanging test were significantly positively the relative abundances of *Lactobacillus*

To confirm the inflammatory process among the different groups, we first measured the corresponding mRNA expression of inflammatory cytokines in the striatum. The results displayed a remarkable upregulation in the MPTP group compared to the control group (MPTP vs. control: *IL-6*, *p* = 0.0131; *IL-1β*, *p* = 0.0182; *Aif1*, *p* = 0.0132; *S100b*, *p* = 0.0132) **(**Fig. 10A**)**. However, the levels in the C.R/MPTP group were even higher than those in the MPTP group (C.R/MPTP vs. MPTP: *IL-6*, *p* = 0.0001; *IFN-γ*, *p* = 0.0055; *S100b*, *p* = 0.0379; *iNOS*, *p* = 0.0486; *CD86*, *p* = 0.0039; *CD68*, *p* = 0.0047; *COX2*, *p* = 0.0293) **(**Fig. 10A **and B)**. However, the mRNA level of *BDNF* was lower in the C.R-, MPTP- and C.R/MPTP groups than the control group (C.R vs. control: *p* = 0.0051, MPTP vs. control: *p* = 0.0361, C.R/MPTP vs. control: *p* = 0.0003) (Fig. S7). Importantly, there was much lower expression in the C.R/MPTP group than the MPTP group (C.R/MPTP vs. MPTP: *p* = 0.0248) (Fig. S7). In addition, the expression of one representative proinflammatory factor (COX2) was measured by western blotting. COX2 protein expression was notably greater in the MPTP group than in the control group (MPTP vs. control: *p* = 0.0241). Compared to that in the MPTP group, the level of COX2 in the C.R/MPTP group was further increased (C.R/MPTP vs. MPTP: *p* = 0.0417), while the COX2 level in the C.R group remained unchanged **(**Fig. 10C**)**. Additionally, the TLR4 antagonist TAK242 was used to prevent the activation of TLR4, and we found that TAK242 had no effect on striatal DA metabolism (Fig.S8).

To investigate the effect of C.R challenge on MPTP-induced PD pathology, we compared the composition of the gut microbiota between the groups receiving MPTP and C.R plus MPTP treatment at 9 d.p.i using 16 S rRNA sequence analysis. We observed a significant increase in α-Diversity, as assessed by the ACE index, in the C.R. plus MPTP-treated group compared to the MPTP group (MPTP vs. C.R/MPTP: *p* = 0.0109) (Fig. S9A). However, no significant differences were found in the Shannon index between the groups (Fig. S9B). PCoA and Bray-Curtis dissimilarity revealed a distinct gut microbiota composition for C.R/MPTP-treated mice compared to MPTP-treated mice (Fig. S9C-D). Furthermore, to identify the specific bacteria impacted by C.R colonization in MPTP-treated mice, we conducted a comparative analysis of microbial relative abundances at various taxonomic levels. At the phylum level (Fig. S9E), the abundance of Bacteroidota and Firmicutes showed a significant decline in the C.R/MPTP group compared to the C.R group, whereas Proteobacteria, in contrast, exhibited a higher abundance in the C.R/MPTP group than the MPTP group (Fig. S9E). At the family level, as illustrated in the pie chart (Fig. S9F), the abundances of Citrobacter, Parabacteroides, Marvinbryantia, and Blautia were higher in the C.R/MPTP group than the MPTP group, while the levels of Muribaculaceae, Lactobacillus, and Lachnospiraceae were lower in the MPTP group. As shown in Fig. S9G, significant differences between the groups were also detected at the genus level. To present detailed information, we selected representative genera and used boxplots (Fig. S9H-K). Compared to the MPTP group, the relative abundance of *Lactobacillus* (*p* = 0.0399) (Fig. S9H) and *Lachnospiraceae* (*p* = 0.0427) (Fig S9I) was notably decreased in the C.R/MPTP group whereas the relative abundance of *Citrobacter* (*p* < 0.0001) (Fig. S9J) and *Parabacteroides* (*p = 0.0015*) (Fig. S9K) showed a significant increase following C.R plus MPTP treatment. These results collectively demonstrated a significantly altered microbiota profile in C.R/MPTP-treated mice.

To gain deeper insights into the role of the microbiota-gut-brain axis in the pathogenesis of PD associated with C.R infection, we conducted correlation analysis on a series of experimental data. Our findings revealed a significant inverse correlation between C.R colonization in the colon and DA level in the striatum (*r* = -0.6645, *p* = 0.0361) **(**Fig. 10E**)**. A positive correlation was observed between fecal calprotectin expression and TLR4 protein expression in the striatum (*r* = 0.9387, *p* = 0.0017) **(**Fig. 10F**)**. Furthermore, a significant negative correlation was identified between the ZO-1 integrity score and the number of TLR4^+^ cells in the SN (*r* = − 0.7947, *p* = 0.0327) **(**Fig. 10G**)**. Additionally, a strong negative correlation was found between the relative abundance of *Parabacteroides* and the number of TH^+^ neurons in the SN (*r* = − 0.8272, *p* = 0.0217) **(**Fig. 10H**)**. The study also revealed a negative correlation between the relative abundance of *Lachnospiraceae* and the number of Iba1^+^ cells in the striatum (*r* = − 0.8851, *p* = 0.0460) **(**Fig. 10I**)**. Furthermore, a significant positive correlation was observed between the relative abundance of *Lactobacillus* and performance in the wire-hanging test (*r* = 0.9328, *p* = 0.0022) **(**Fig. 10J**)**. Collectively, these correlation results provide strong evidence for the involvement of the microbiota-gut-brain axis in the development of PD, especially when C. R infection is present.

## Discussion

**Fig. 11:**
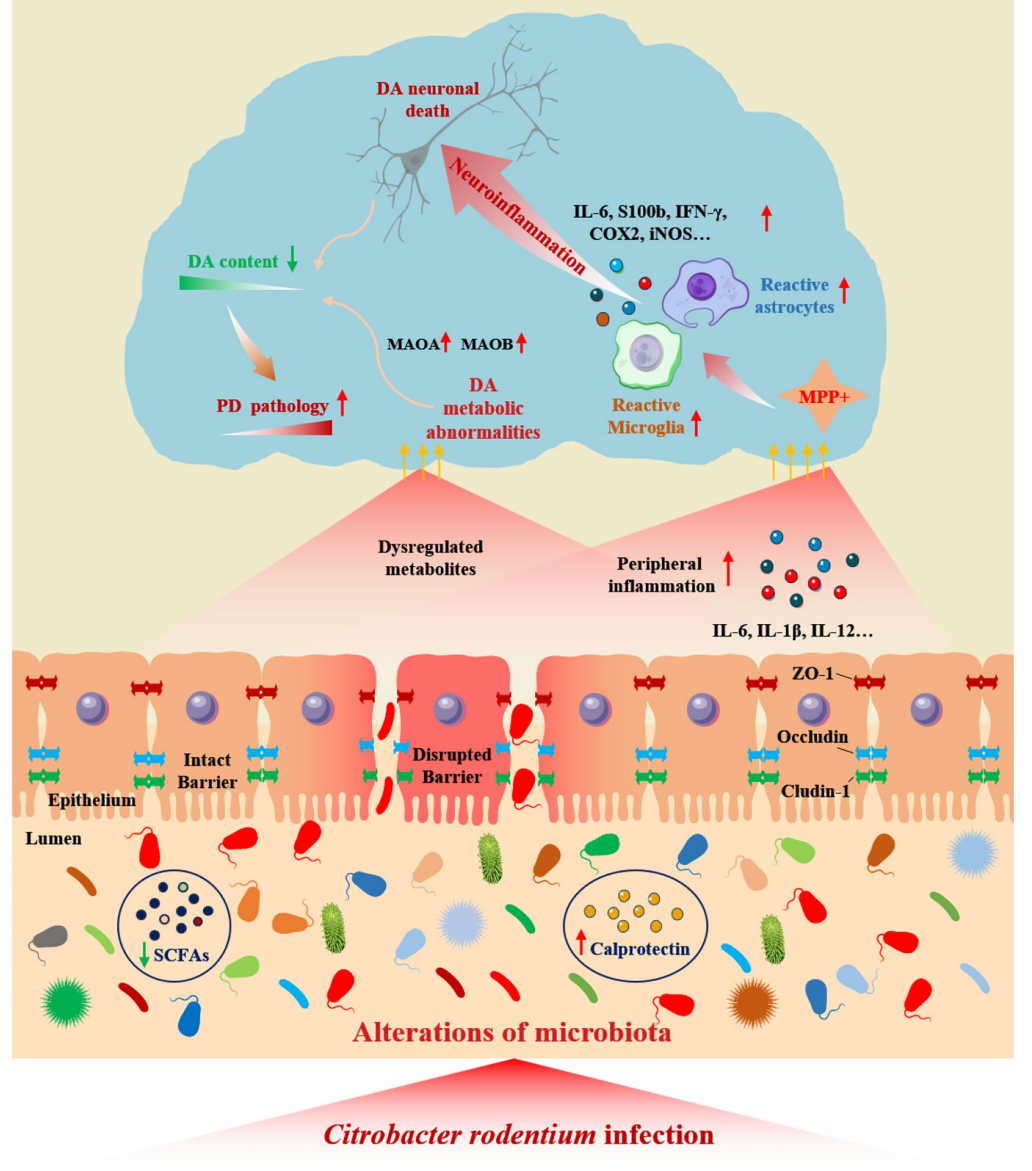
Diagram of C.R infection impairs dopamine metabolism and exacerbates the pathology of Parkinson’s disease

There is increasing evidence that intestinal inflammation is a high risk factor for developing PD [34]. However, the detailed mechanism remains unknown. In the present study, we demonstrated that C.R infection induced an IBD-like pathology. Notably, neurotransmitter metabolism was also affected by C.R infection. Additionally, C.R infection further exacerbated MPTP-induced motor deficits, the impairment of the nigrostriatal dopaminergic system, glial cell activation and neuroinflammation in mice (Fig. 11).

C.R infection, which closely mimics IBD-like disease, is widely used to establish a murine infection model [21]. After the animals were infected with C.R, astrocytes were activated in the colon plexuses, similar to the findings in another colitis model [35]. Many inflammatory markers in the C.R-challenged colon displayed increased expression. In addition, the level of calprotectin was significantly increased in the feces of the C.R-infected mice, consistent with the findings in IBD patients [36], suggesting the development of intestinal inflammation after C.R colonization.

Intestinal inflammation also affects intestinal barrier function [37]. In agreement with a previous report, we observed that the C.R challenge caused a significant increase in FD4 intensity in the serum. H&E staining of the colon tissues revealed an immune infiltration and epithelial damage caused by C.R infection. In addition, our work showed that the expression of key tight junction- proteins (ZO-1, Occludin, and Claudin-1) in colonic tissues was markedly decreased after C.R colonization. Moreover, at the mRNA level, nine tight junction molecules in the colonic tissues were analyzed, and consistent results was obtained. These phenomena suggested increased colonic permeability [35, 38, 39].

C.R infection impacts the intestinal microbiota composition [40, 41]. The α-Diversity of the gut microbiota significantly decreased while the microbial diversity did not change in C.R-infected mice, which was consistent with previous studies [19]. At the moment, the C.R strain plausibly exhibited dominance and engaged in competition with other microorganisms for resources and habitat, consequently impacting microbial abundance rather than diversity. However, unlike other reports, C.R infection greatly disturbed bacterial communities as indicated by β-Diversity in the present data [19]. This discrepancy may be due to differences in the genetic background, feeding conditions and diets of the mice [19, 41]. At the phylum level, in accordance with the observations in PD patients, C.R challenge significantly reduced the relative abundance of *Firmicutes* and increased the levels of *Bacteroidetes*, *Proteobacteria*, *Verrucomicrobia* and *Epsilonbacteraeota* [42]. It has been reported that increases in *Proteobacteria* can be a consequence of gut inflammation [43]. Notably, the ratio of *Bacteroidetes* to *Firmicutes* increased which was associated with increased susceptibility to C.R [44]. At the family level, the relative abundance of *Enterobacteriaceae* increased after the C.R challenge, which is similar to observations in PD patients, where this increase in *Enterobacteriaceae* abundance was positively associated with the severity of postural instability and gait difficulty [45]. At the genus level, we noted a modest increase in *Akkermansia* abundance after challenge with C.R; *Akkermansia* is a gram-negative mucin-degrading microbe associated with anti-inflammatory immune signatures in humans [46]. *Akkermansia* considered a signature of the fecal microbiota of PD patients compared to that of healthy individuals [47]. A greater proportion of *Akkermansia* was also observed in a chronic stress-induced mouse model of PD [35]. Additionally, increased levels of *Helicobacter pylori* and *Roseburia* have also been reported to be associated with PD [42, 48]. The abundances of *Ruminococcus* and *Parasutterella*, which are closely associated with Crohn’s disease, are obviously increased, which was in consistent with our data [49, 50].

Gut microbiota disorders, such as alterations in SCFAs, cause metabolic abnormalities in the gut. One of the *Firmicutes*, *Lachnospiraceae* which can produce SCFAs showed a significant decrease in abundance in C.R-infected animals [51]. Acetate, propionate, and butyrate are the primary SCFA molecules produced from gut bacterial fermentation and are endowed with anti-inflammatory properties. In fecal samples from C.R-infected mice, the levels of butyric acid and propionic acid were prominently decreased, in line with the findings in PD patients, while the acetic acid level remained unchanged, which was different from the findings that observed in PD patients [52]. C.R infection caused obvious intestinal inflammation, which may be related to the abnormal metabolism of SCFAs. In addition, the levels of GRP109a and GPR43, which mainly respond to SCFAs, were significantly decreased after the C.R challenge, further supporting the insufficiency of SCFAs in the gut. In addition, SCFAs are highly implicated in intestinal barrier integrity and intestinal permeability [53]. Within our model system, intestinal barrier impairment and high permeability were observed after the C.R challenge, and SCFAs such as propionic acid and butyric acid were altered, which may be a plausible underlying mechanism [54, 55].

Gut microbiota disorders affect neurotransmitter metabolism in the brain. After the administration of C.R, we found that the levels of DA and 5-HT in the striatum decreased with increasing levels of the downstream metabolites DOPAC, HVA, and 5-HIAA, respectively, with no differences between the sexes, suggesting increased turnover of dopamine and 5-HT. The rate of turnover recovered at 30 days post infection, and the complete clearance of C.R in the gut may also be present at this timepoint [28]. The decrease in DA may be partially attributed to the upregulation of MAOA and MAOB, which are two critical enzymes involved in the metabolism of DA [56, 57].

We chose a single high-dose MPTP to induce PD-like pathology, and we observed dopaminergic degeneration, which was consistent with previous reports. However, the motor behaviors of MPTP-treated mice were unchanged compared to those of normal saline-treated mice 3 days after neurotoxicity. In accordance with earlier reports, the behavioral performance in our study may return to normal in mice 3 days after MPTP administration [58, 59]. C.R infection potentiated the MPTP-induced loss of dopaminergic neurons in the SN and dopaminergic fibers in the striatum. Consequently, we were able to observe that animal behavioral performance appeared to be most sensitive to the combined effects of C.R and MPTP, suggesting that the sensorimotor system could be highly impacted by the gut-originated proinflammatory state, what has been observed in other studies [13, 60, 61]. C.R challenge alone had no obvious effect on the movement performance of the animals, consistent with the findings of other studies [20]. However, inconsistent with one report, in the present study, anxiety-like behavior was observed at 9 days after C.R infection [62]. The mood disorders may be attributed to the dysfunction of 5-HT systems. We found there was abnormal metabolism of 5-HT in the C.R-infected mice, and a similar phenomenon was also reported in a depression model [63].

In the current study, C.R infection potentiated the MPTP-induced effects on microglial and astrocyte activation in the nigrostriatal pathway. First, the combination of C.R and MPTP changed the state of microglia from “ramified” to “stouter”, and polarized microglia to the M1 proinflammatory phenotype (manifested by the excessive increase in CD16/32-positive cells) and astrocytes to the A1 phenotype (demonstrated by the large increase in C3-marked astrocytes). Second, there were increased expression levels of proinflammatory molecules (*IL-6*, *IFN-γ*, *S100b*, *iNOS*, *CD86*, *CD68* and *COX2*) in the striatum of the C.R/MPTP mice. The expression of COX2 protein was also upregulated. Third, the ELISA results showed increased serum levels of the cytokine IL-6. These data clearly confirmed that C.R infection exacerbated the MPTP-mediated inflammatory response. The concept of immune training has been proposed by previous studies showing that peripherally applied inflammatory stimuli can induce acute immune training in the brain, which effectively acts as an important modifier of neuropathology [64]. Our results implied that there may be an immune training response to preestablish C.R infection in the brain. The precise mechanism of immune memory in response to bacterial stimulation still needs to be confirmed in future studies.

C.R infection exacerbated MPTP-induced PD pathology at the gut microbiota level in the present study. Cotreatment with C.R. and MPTP markedly decreased the relative abundance of the Bacteroidetes and Firmicutes phyla while increasing the abundance of Proteobacteria. At the family level, there was a noticeable increase in the relative abundance of Citrobacter and Parabacteroides, suggesting a significant presence of intestinal inflammation. Furthermore, the combination of MPTP and C.R resulted in a significant reduction in the relative abundance of *Lactobacillus* and *Lachnospiraceae* genera, both of which are known for producing SCFAs [51]. However, this combination treatment led to a modest increase in the abundance of *Citrobacter* and *Parabacteroides*, which are gram-negative bacteria characterized by a high abundance of LPS on their outer membrane [51, 65]. LPS can act as a stimulator of the TLR4 pathway, potentially contributing to the exacerbation of PD pathology. According to our data, TLR4 expression was indeed upregulated in the colon and brain after C.R and MPTP combined treatment. Furthermore, intestinal barrier damage and high permeability were observed in the colon after C.R delivery. This finding prompted us to speculate that bacteria and/or bacterial molecules might leak into the circulation. This leakage contributes to systemic inflammation, leading to BBB disruption and neurodegeneration in the central nervous system. Herein, we hypothesized that certain molecules, such as LPS, might reach the SN and striatum regions and locally stimulate the TLR4 pathway, thus initiating or increasing the expression of proinflammatory cytokines. Therefore, the TLR4-NF-κB signaling pathway might be involved in our model system. However, further in-depth studies are necessary to verify the significance of this signaling axis. To further validate the significance of the microbiota-gut-brain axis in our study, we performed correlation analysis on various representative results. The alterations in the gut microbiota caused by C.R. infection were closely associated with the concentrations of DA, the expression of TLR4, the activation of microglia, and the density of dopaminergic neurons in the nigrostriatal pathway. These strong correlations suggest that microbiota disorders induced by C.R. infection play a crucial role in the pathogenesis of PD through the microbiota-gut-brain axis.

## Conclusions

In conclusion, our study further elucidates how C.R infection affects PD. One mechanism is that C.R infection-induced microbiota imbalance disturbs dopamine metabolism, particularly its turnover rate. The other mechanism is that the IBD-like symptoms caused by C.R infection exacerbate MPTP-induced PD pathology in mice. The IBD-like model induced by C.R infection is an ideal model for studying the relationship between intestinal inflammation and PD.

### Electronic supplementary material

Below is the link to the electronic supplementary material.

Supplementary Material 1

## Data Availability

The data and materials generated during the current study are not publicly available but are available from the corresponding author upon reasonable request.

## Abbreviations

C.R: Citrobacter Rodentium
IBD: Inflammatory Bowel Disease
UC: Ulcerative Colitis
CD: Crohn’s Disease
SCFAs: Short Chain Fatty Acids
BBB: Blood-brain Barrier
LPS: Lipopolysaccharide
SN: Substantia Nigra
DSS: Dextran Sulphate Sodium
CFUs: Colony-forming Units
RT-qPCR: Real-time Quantitative PCR
C3: Complement Component 3
GI: Gastrointestinal Dysfunctions
DA: Dopamine
D.p.i: Days Post Infection
MAOA: Monoamine Oxidase A
MAOB: Monoamine Oxidase B
