# Supplementary material for: Citrobacter rodentium infection impairs dopamine metabolism and exacerbates the pathology of Parkinson’s disease in mice
Source: J Neuroinflammation. 2024 Jun 7;21:153. doi: 10.1186/s12974-024-03145-0 (PMC11161935; doi:10.1186/s12974-024-03145-0)
Supplement: Supplementary file 1 — Supplementary Material 1 [file 12974_2024_3145_MOESM1_ESM.docx]

**Supplement materials**

**Table S1** The primer for the RT-qPCR.

| **Gene name** | **Forward oligonucleotide (5’-3’)** | **Reverse oligonucleotide (5’-3’)** |
| --- | --- | --- |
| Ocln | CCCCGGAAGATCGTGTTTG | TAGTCAGGTACTGGGTTGAGG |
| Cldn3 | ACCAACTGCGTACAAGACGAG | CAGAGCCGCCAACAGGAAA |
| Cldn4 | GCATGGTGGGAACGCTCAT | CCACAGTCCCTTCAGGTAGGA |
| Cldn7 | CTGGAGGCATTGTTTTCATTGTG | CATGGGCGTCAAGGGGTTA |
| Cldn8 | GCAACCTACGCTCTTCAAATGG | TTCCCAGCGGTTCTCAAACAC |
| Cldn12 | TGTCCTTCCTGTGTGGTATTGC | AAATCGTCAGGTTCTTCTCGTTT |
| Tjp1 | GCTTTAGCGAACAGAAGGAGC | TTCATTTTTCCGAGACTTCACCA |
| Tjp2 | ATGGGAGCAGTACACCGTGA | TGACCACCCTGTCATTTTCTTG |
| Tjp3 | TCGGCATAGCTGTCTCTGGA | GTTGGCTGTTTTGGTGCAGG |
| TNF-α | CGTCAGCCGATTTGCTATCT | CGGACTCCGCAAAGTCTAAG |
| iNOS | CCCTTCCGAAGTTTCTGGCAGCAGC | GGCTGTCAGAGCCTCGTGGCTTTGG |
| IL-12 | GTGGACCAAACAATCTGACCTG | AACACGGACTATGAACCTGGA |
| IL-1β | GAGAGCCGGGTGACAGTATC | TGACAAACTTCTGCCTGACG |
| IL-4 | GGTCTCAACCCCCAGCTAGT | GCCGATGATCTCTCTCAAGTGAT |
| IL-6 | CCTACCCCAATTTCCAATGCT | TATTTTCTGACCACAGTGAGGAAT |
| IFN-γ | CTGGCAGGATGATTCTGCTGG | GCATACGACAGGGTTCAAGTTAT |
| Aif1 | ATCAACAAGCAATTCCTCGATGA | CAGCATTCGCTTCAAGGACATA |
| MHCⅡ | AGCATCCAAGGAACCCACAC | CAGCATCCCAAGGGCTCTG |
| S100b | TGGTTGCCCTCATTGATGTCT | CCCATCCCCATCTTCGTCC |
| CD86 | TGTTTCCGTGGAGACGCAAG | TTGAGCCTTTGTAAATGGGCA |
| CD68 | CCATCCTTCACGATGACACCT | GGCAGGGTTATGAGTGACAGTT |
| COX2 | GTTCATCCCTGACCCCCAAG | ACTCTGTTGTGCTCCCGAAG |
| TLR4 | ATGGCATGGCTTACACCACC | GAGGCCAATTTTGTCTCCACA |
| GPR43 | GGCTCAGAAGCAAGGTGACT | TGTGGCTTAGAGCTTTCCCG |
| GPR109a | GGAATGCCGGGAAAGCTCTA | AAGCATGTGACCCAGATCCC |
| BDNF | TCATACTTCGGTTGCATGAAGG | AGACCTCTCGAACCTGCCC |
| β-actin | CAGGATGCAGAAGGAGATTAC | AACGCAGCTCAGTAACAGTC |

**Table S2** The treatment combinations and times in the study.

| **Purpose of experiment** | **Treatment methods** | **Treatment time** |
| --- | --- | --- |
| 1. Analysis of the gut microbiome | C.R treatment | For 9 days |
| 2. Evaluate the intestinal barrier damages and intestinal inflammation | C.R treatment | For 9 days |
| 3.Measure the intestinal transmission distance and colon length | C.R treatment | For 9 days |
| 4.Metabolic abnormality of SCFAs in the gut | C.R treatment | For 9 days |
| 5. Metabolic abnormality of neurotransmitters in the striatum | C.R treatment | For 9, 15, 21, 30 days |
| 6. Evaluate the effects of C.R infection on MPTP-induced motor deficits | C.R plus MPTP treatment | For 9 days |
| 7. Evaluate the effects of C.R infection on MPTP-induced on the dopaminergic destruction | C.R plus MPTP treatment | For 9 days |
| 8. Evaluate the activation of microglia and astrocyte in the striatum | C.R plus MPTP treatment | For 9 days |
| 9. Evaluate the TLR4 signaling pathway in the colon and in the striatum | C.R plus MPTP treatment | For 9 days |
| 10. Evaluate the neuroinflammation in the striatum | C.R plus MPTP treatment | For 9 days |


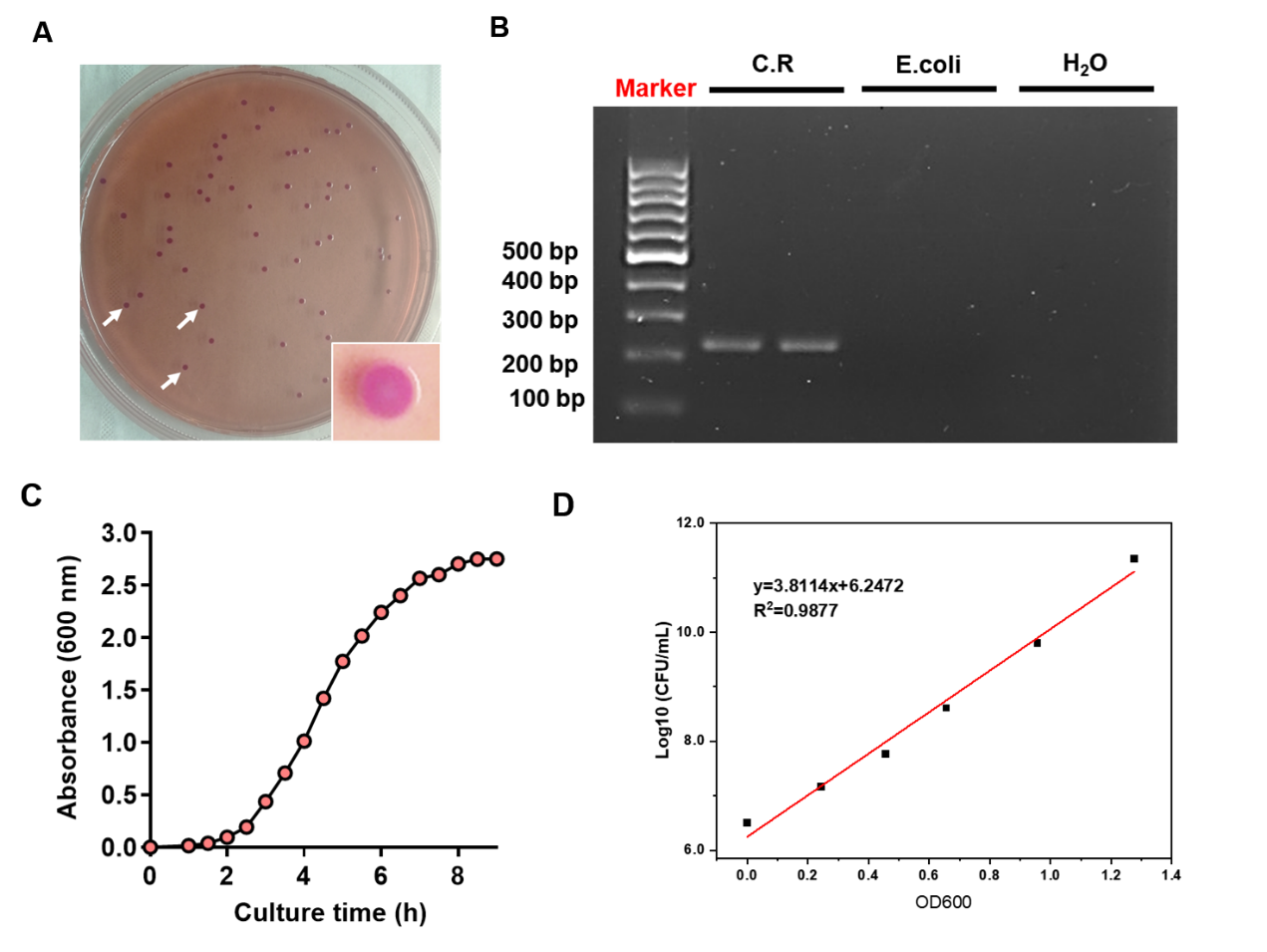


**Fig. S1** C.R culture. (A) C.R was cultured in chloramphenicol MacConkey agar plates for 24 h. The arrow indicated the mono-clone colony. (B) DNA extracted from *E. coli* and C.R was amplified by PCR using bacteria-specific primers. The H_2_O served as the control. (C) Culture time of C.R relative to the absorbance (OD600) within 8 h. (D) The linear relationship between the absorbance (OD600) and the bacterial concentration.


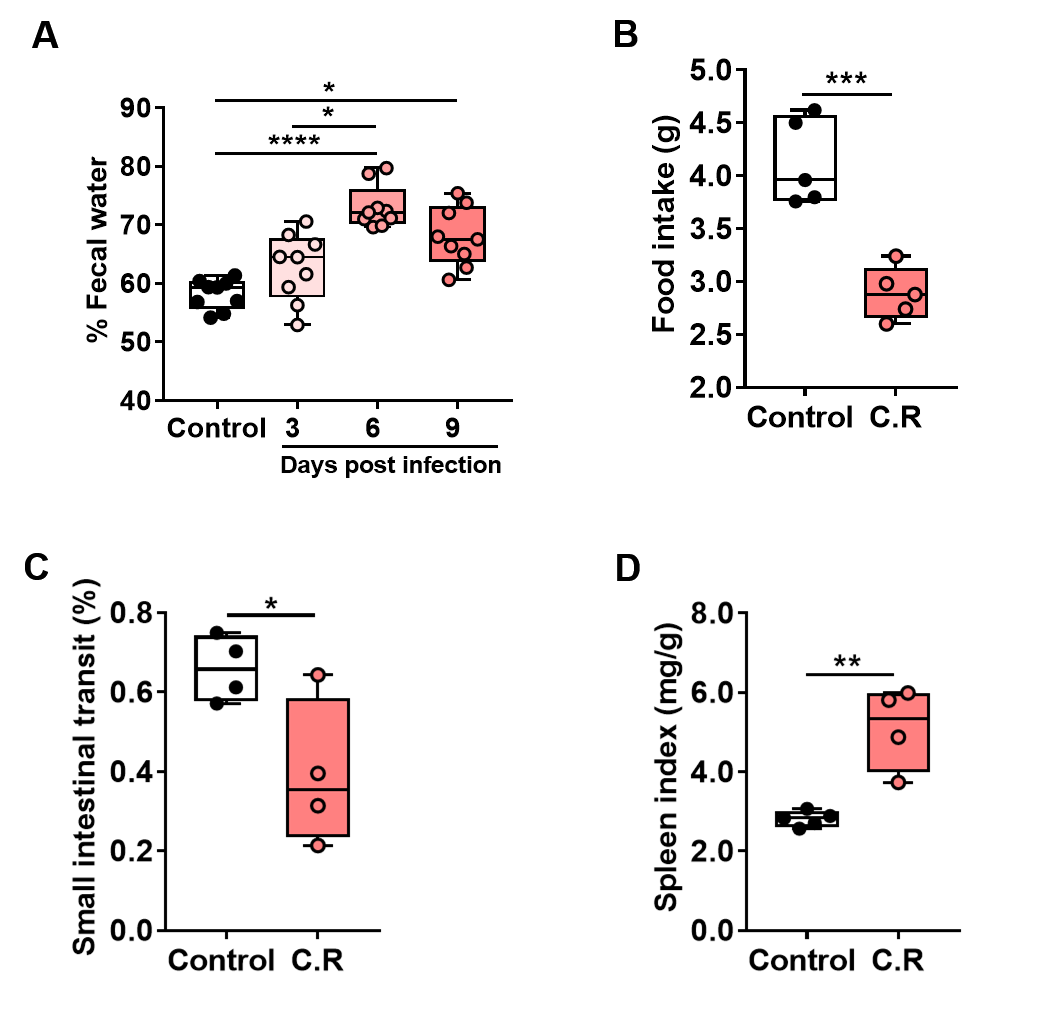


**Fig. S2** Pathological index analysis. **(A)** Water content in feces was analyzed at 3-day intervals after infection with C.R. n = 9. **(B)** Food consumption was analyzed at 9 d.p.i.. n = 5. **(C)** Distance of small intestinal transit was analyzed at 9 d.p.i.. n = 4. **(D)** Spleen index (spleen weight per body weight) was analyzed at 9 d.p.i.. n = 4-5. **p* < 0.05, ***p* < 0.01, ****p* < 0.001,*****p* < 0.0001.


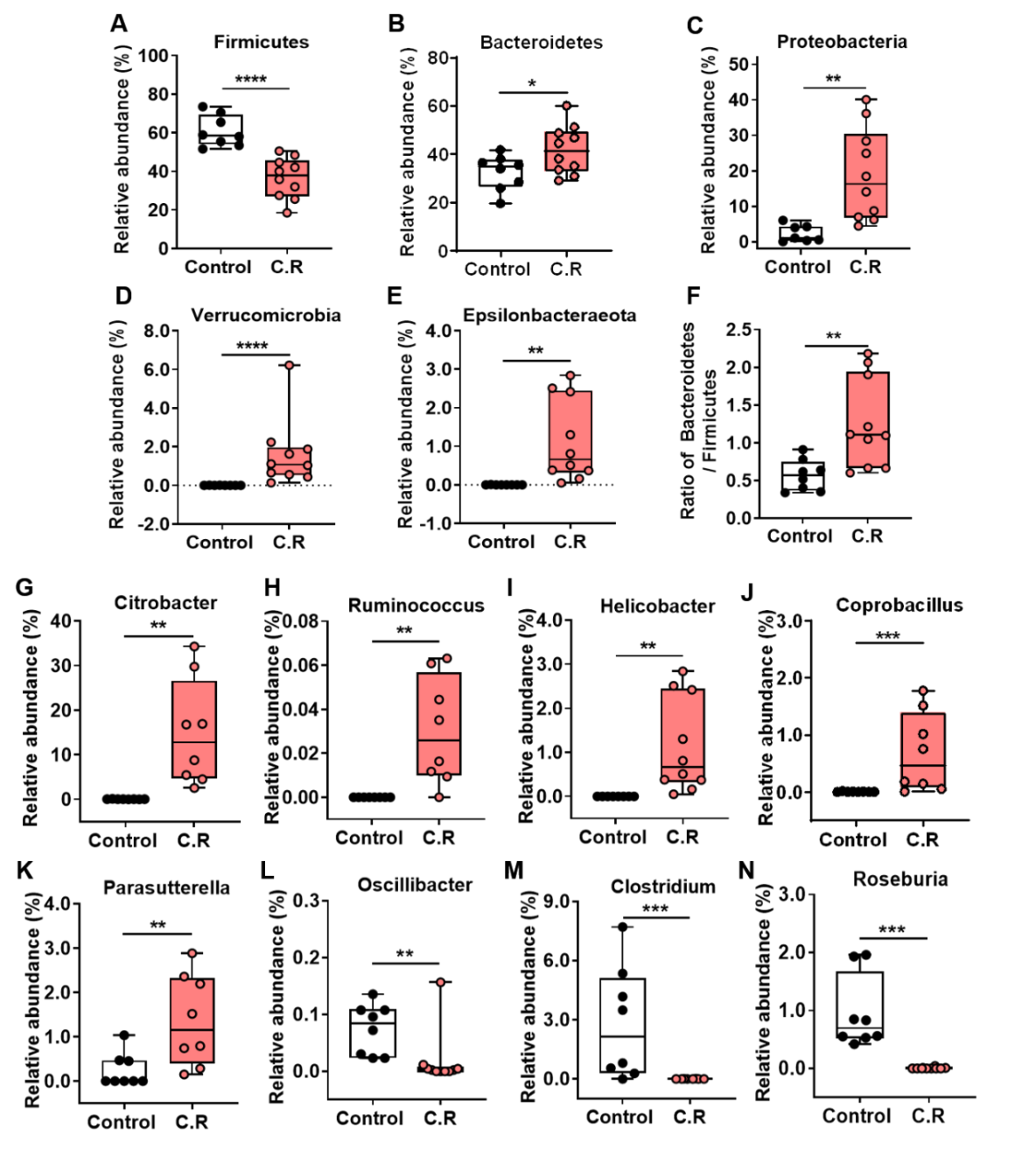


**Fig. S3** Analysis of the gut microbiome at the phylum level and the genera level. **(A-E)** Relative abundances of 5 significantly altered bacterial phyla: *Firmicutes* **(A)**, *Bacteroidetes* **(B)**, *Proteobacteria* **(C)**, *Verrucomicrobia* **(D)**, *Epsilonbacteraeota* **(E)**. **(F)** The ratio of relative abundances of *Bacteroidetes* to *Firmicutes* at the phylum level. **(G-N)** Relative abundances of 10 significantly altered bacterial genera: *Citrobacter* **(G)**, *Ruminococcus* **(H)**, *Helicobacter* **(I)**, *Coprobacillus* **(J)**, *Parasutterella* **(K)**, *Oscillibacter* **(L)**, *Clostridium* **(M)**, *Roseburia* **(N)**. n = 8-10. **p* < 0.05, ***p* < 0.01, ****p* < 0.001, *****p*< 0.0001.


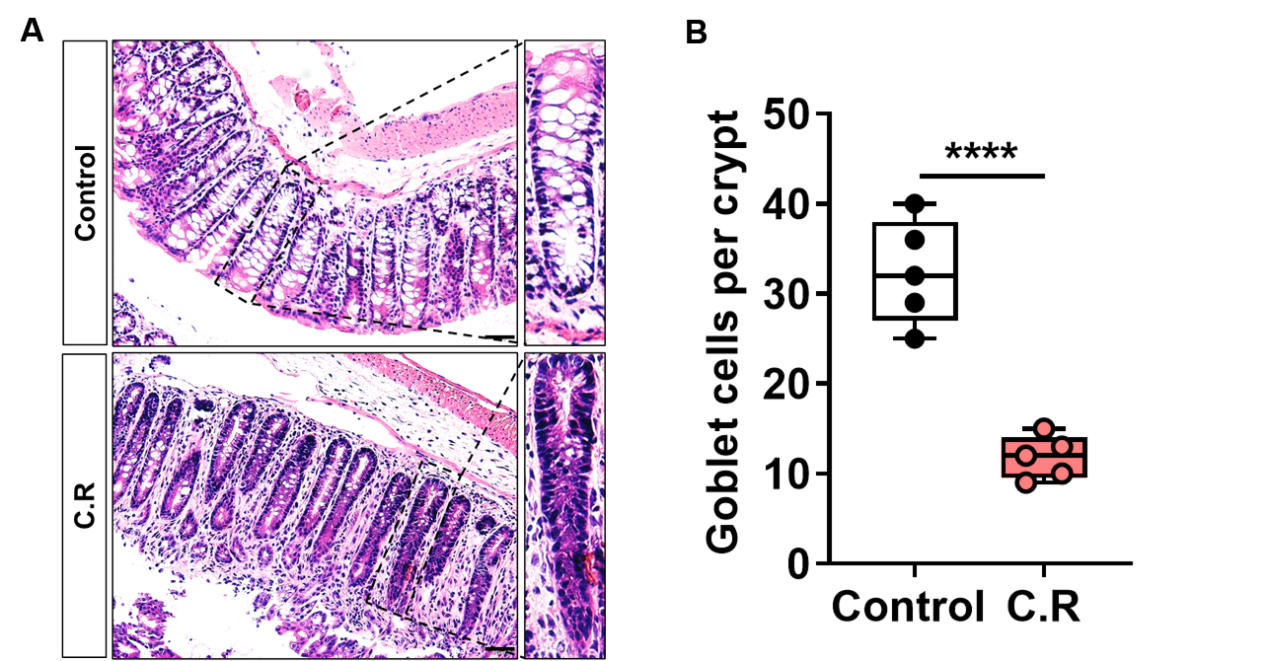


**Fig. S4** C.R infection impacted on the colon. **(A)** H&E staining of colons. Scale bar, 50 µm. **(B)** The number of goblet cells per crypt. n = 5. *****p* < 0.0001.


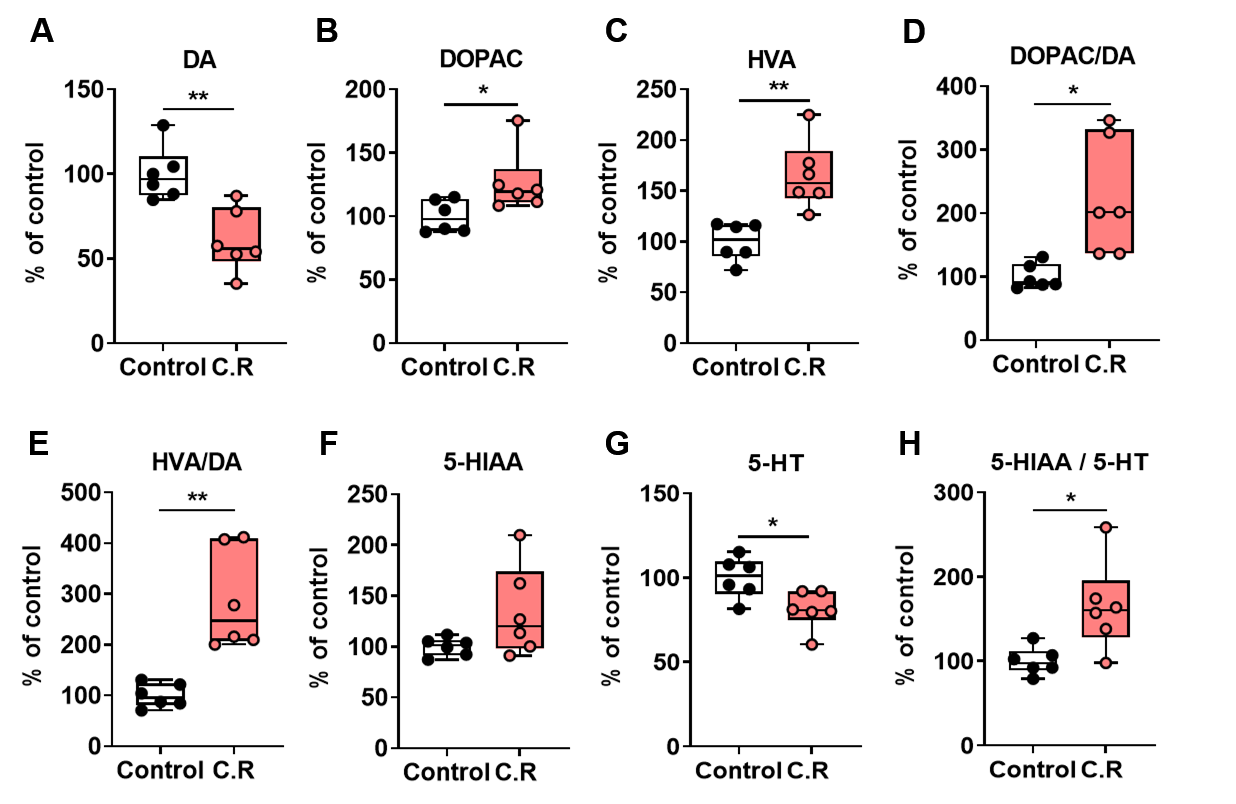


**Fig. S5** C.R infection caused metabolic abnormality of striatal neurotransmitters in the female mice. **(A-H)** The metabolism of DA and 5-HT. DA **(A)**, DOPAC **(B)**, HVA **(C)**, DOPAC/DA **(D)**, HVA/DA **(E)**, 5-HIAA **(F)**, 5-HT**(G)**, 5-HIAA/5-HT **(H)**. n = 5-6. **p* < 0.05, ***p* < 0.01.


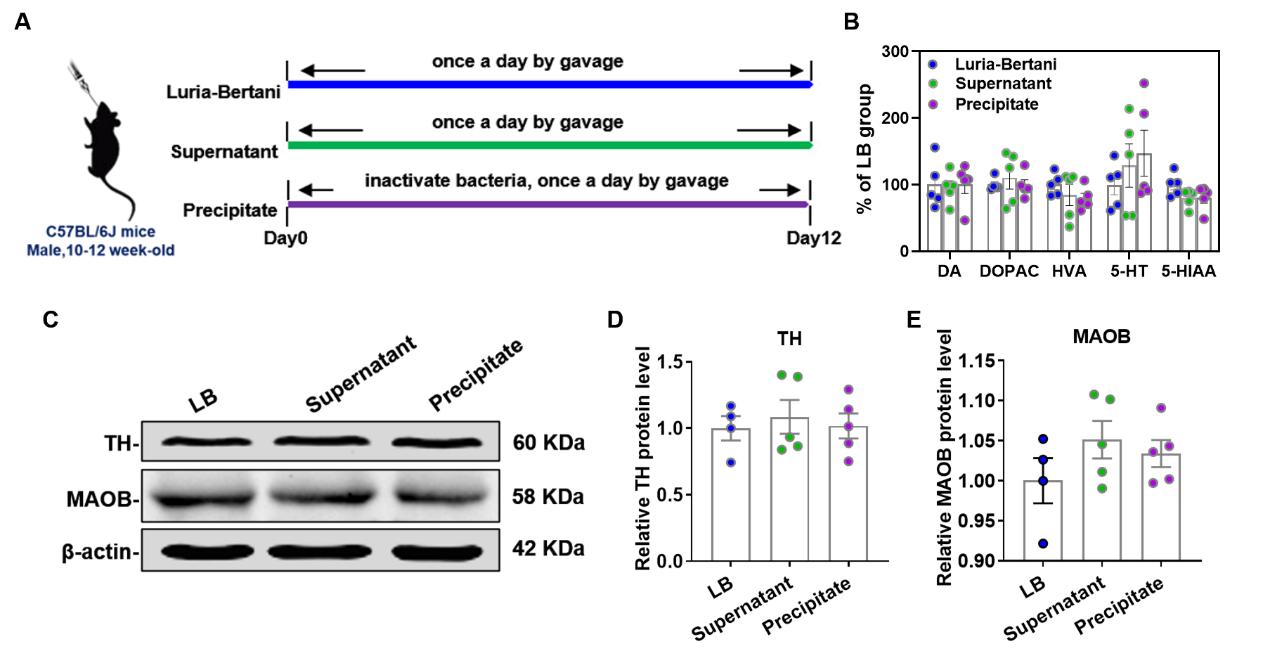


**Fig. S6** Both the supernatant and the precipitate of C.R did not affect the striatal neurotransmitter metabolism, TH and GFAP protein levels. (A) The experimental design in the current study. **(B)** The relative levels of DA, DOPAC, HVA, 5-HT and 5-HIAA in the striatum. n = 5. **(C)** Western blot showing striatal levels of TH and MAOB proteins. β-Actin serves as the loading control. **(D-E)** Quantification of relative expression levels of TH and MAOB proteins. n = 4-5.


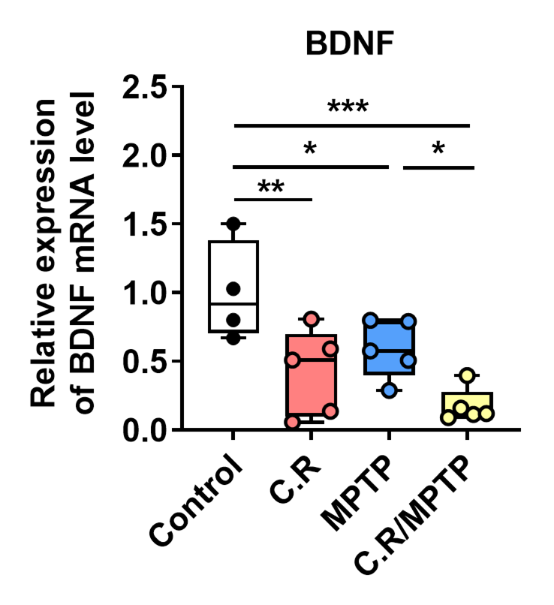


**Fig. S7** The mRNA level of *BDNF* in the striatum was analyzed by qPCR. n = 4-5. **p* < 0.05, ***p* < 0.01, ****p* < 0.001.


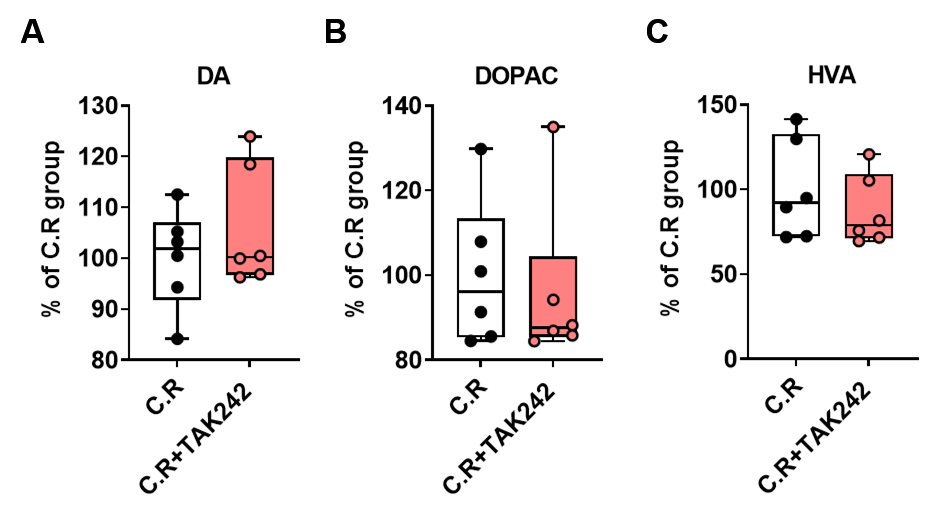


**Fig. S8** TLR4 antagonist TAK242 treatment had no effect on the metabolism of striatal dopamine in the male mice. **(A-C)** The metabolism of DA. DA **(A)**, DOPAC **(B)**, HVA **(C)**. n = 6.


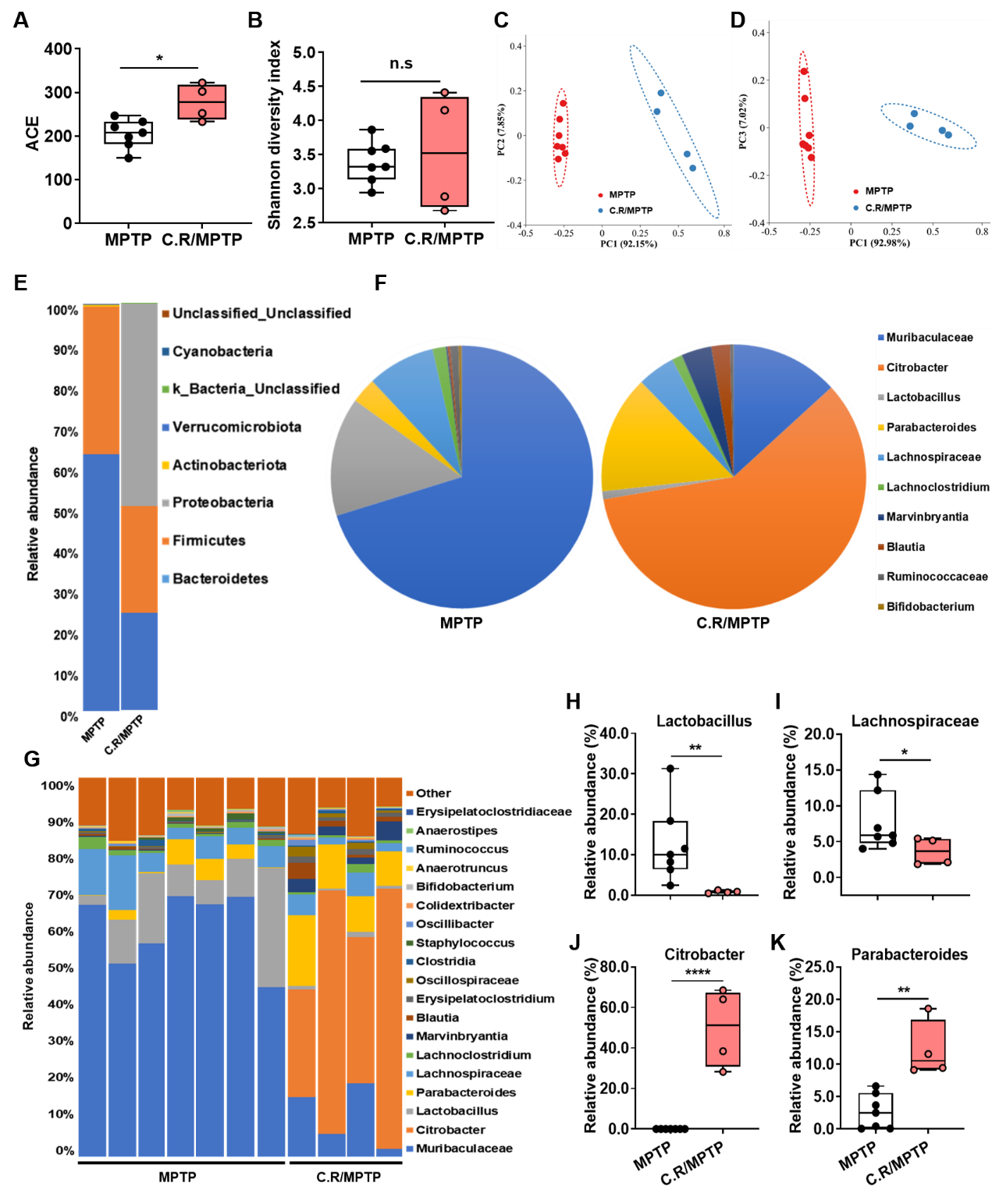


**Fig. S9** Analysis of the gut microbiome in MPTP- and C.R plus MPTP-treated mice at 9 days. **(A-B)** Analysis of α-Diversity based on 16S rRNA sequencing predicted gut microbiota richness by the ACE index **(A)**, and diversity by the Shannon index **(B)**. **(C-D)** PCoA plots were based on weighted UniFrac metrics of gut microbiota where samples of mice from different groups were highlighted with different colors. Principal components (PCs) 1 and 2 explain 92.15% and 7.85% **(C)**, 1 and 3 explain 92.98% and 7.02% **(D)** of the variance, respectively. The position and distance of data points indicated the degree of similarity in terms of both the presence and relative abundance of bacterial taxonomies. **(E)** Percentage stacking chart based on the Bray-Curtis distance analysis about the relative abundance of gut microbiota at the phylum level between the groups. **(F)** Relative abundance of gut microbiota at the family level between the groups. **(G)** Relative abundances of gut microbiota at the genus level between the groups. **(H-K)** Relative abundances of 4 significantly altered bacterial genera: *Lactobacillus* **(H)**, *Lachnospiraceae* **(I)**, *Citrobacter* **(J)**, *Parabacteroides* **(K)**. n = 7 or 4, respectively. **p* < 0.05, ***p* < 0.01, *****p* < 0.0001.

**
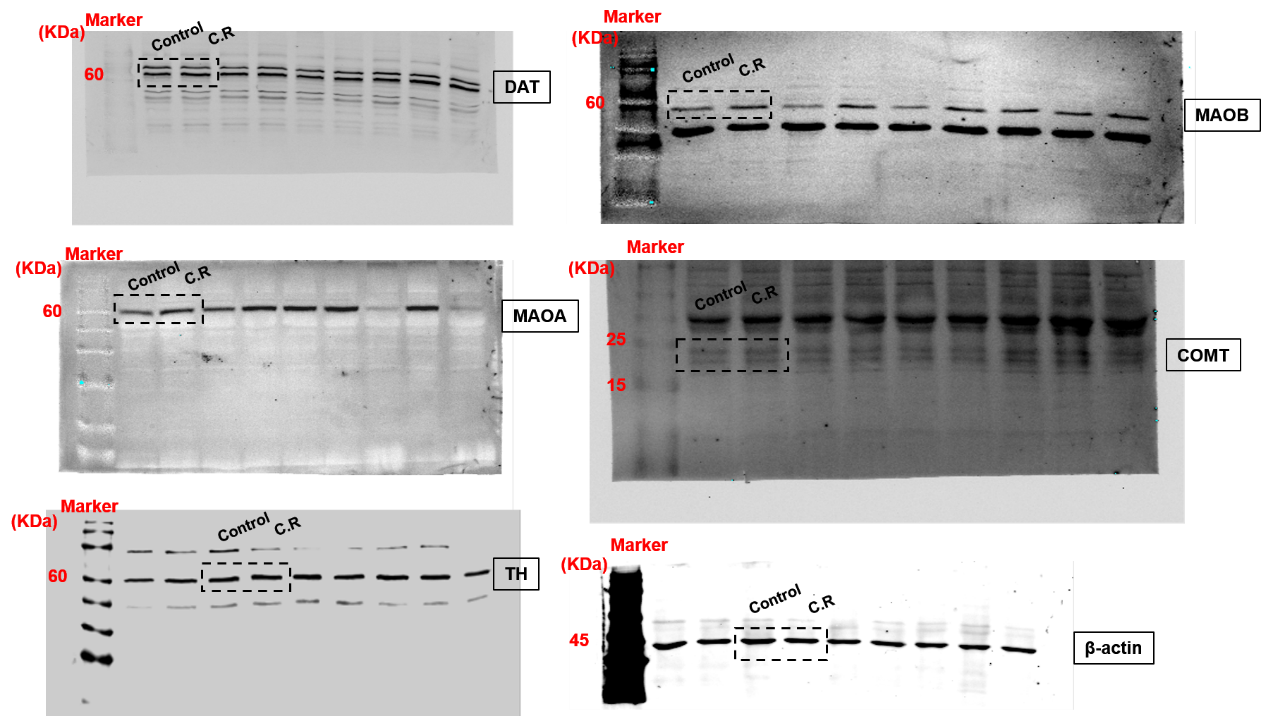
**

**Fig S10.** The original blot of the **Fig.3N**.


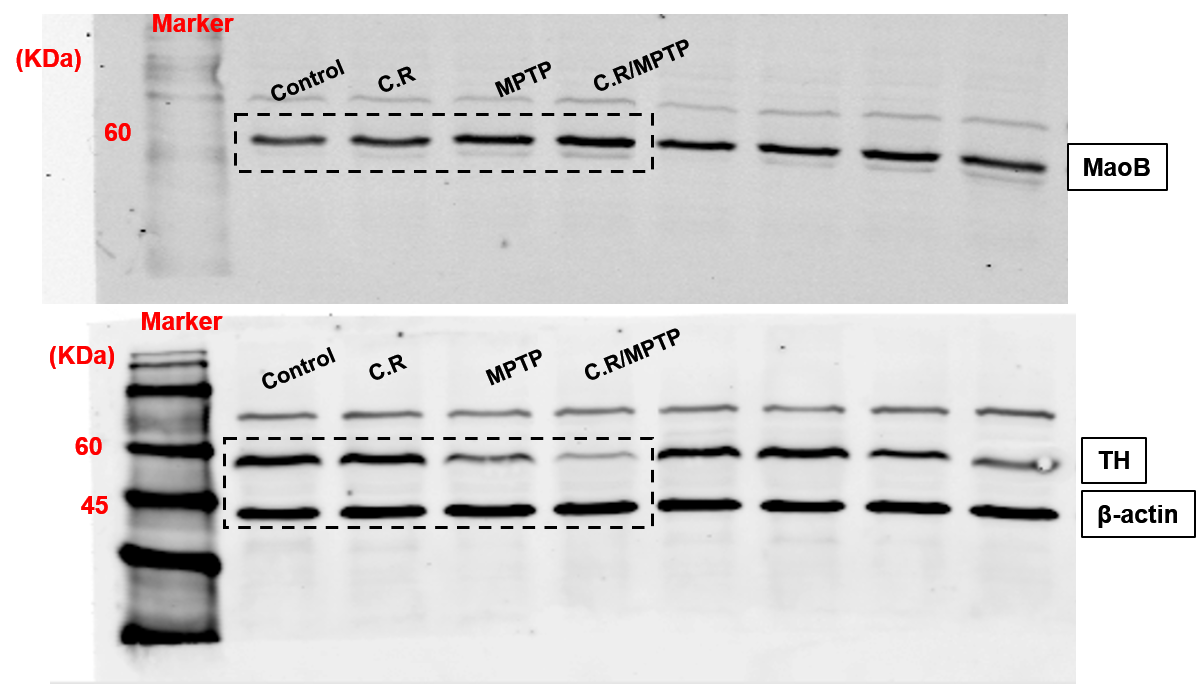
 **Fig S11.** The original blot of the **Fig. 5E**.


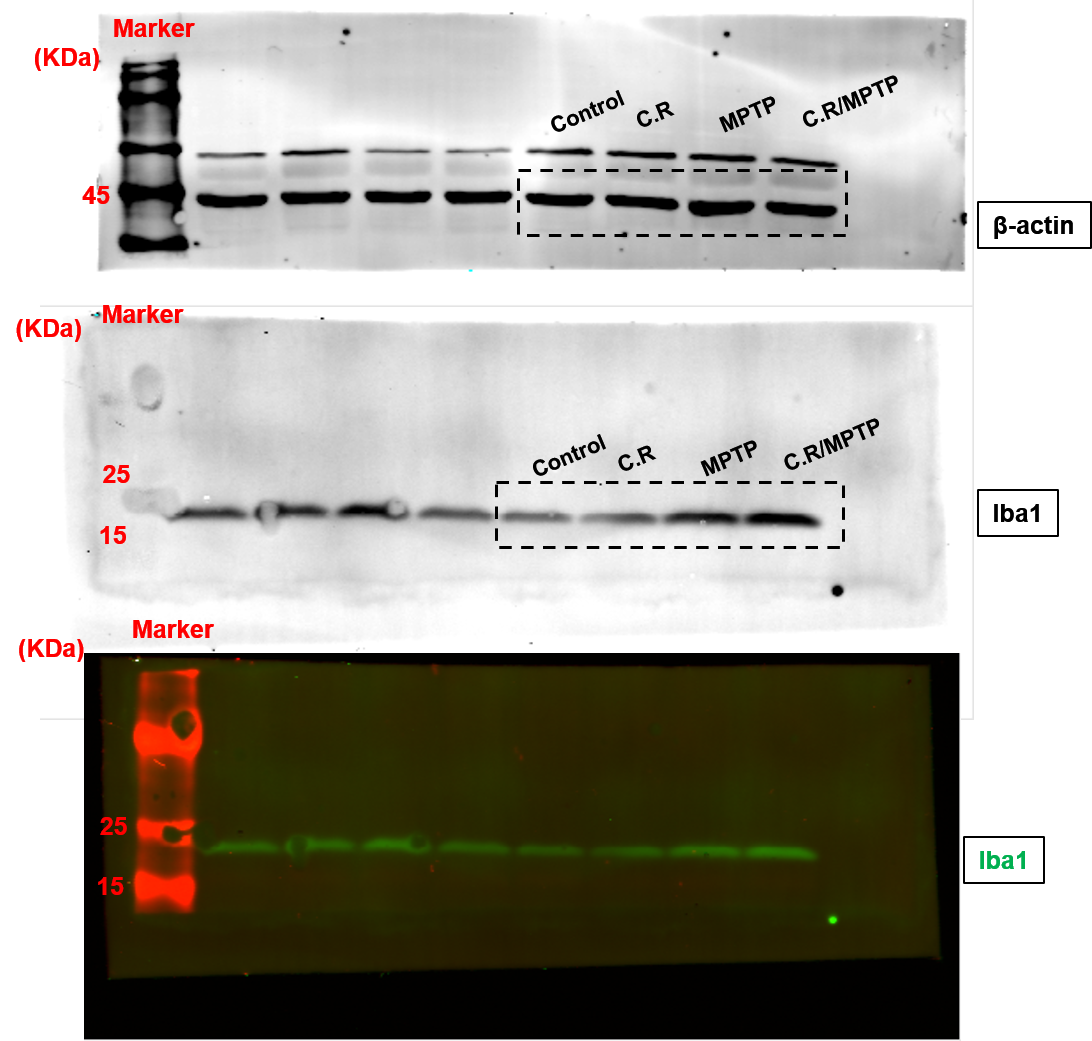
 **Fig S12.** The original blot of the **Fig. 6A**.


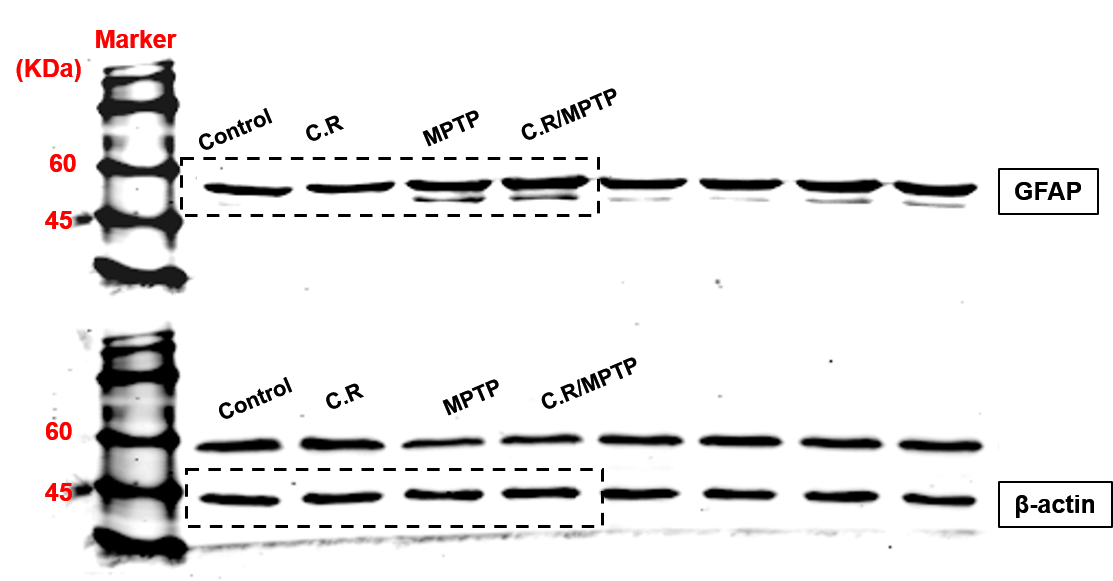
 **Fig S13.** The original blot of the **Fig. 7A**.

**
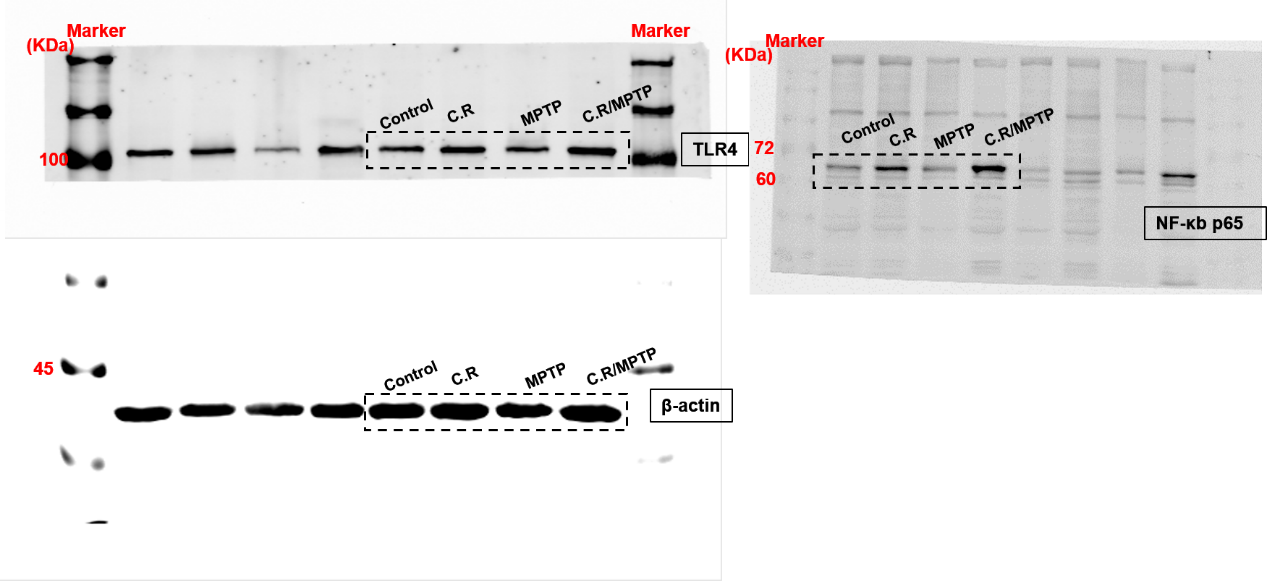
**

**Fig S14.** The original blot of the **Fig. 8B**.


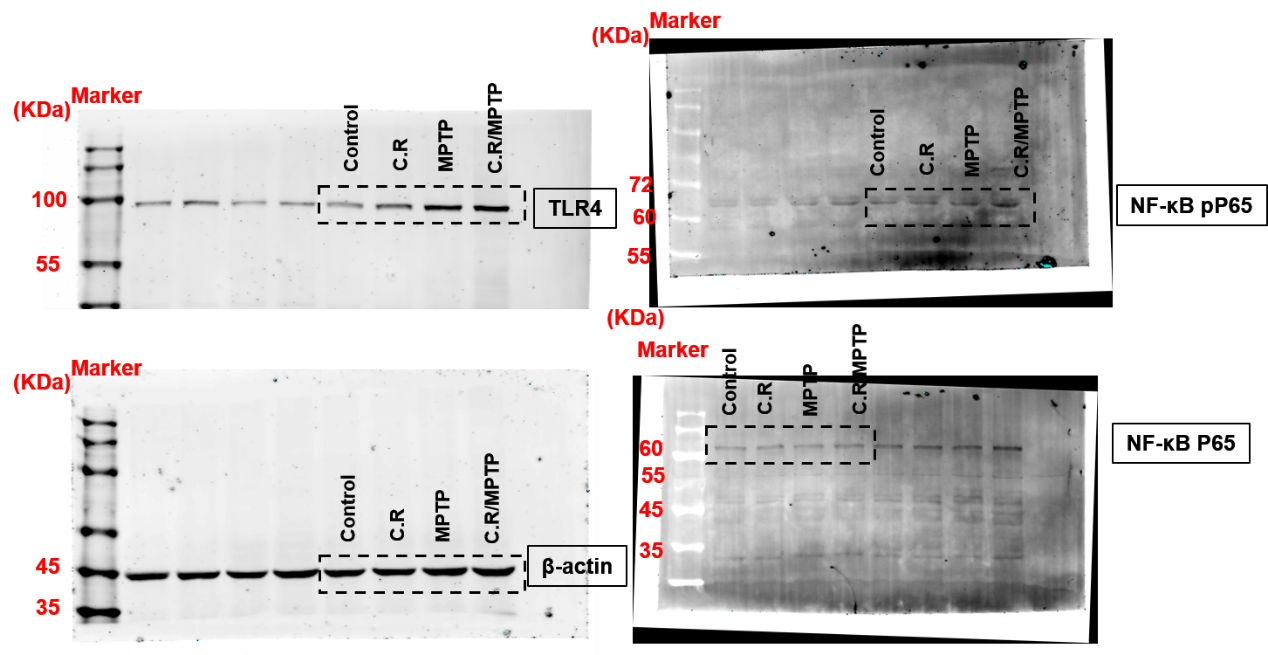


**Fig S15.** The original blot of the **Fig. 9A**.

**
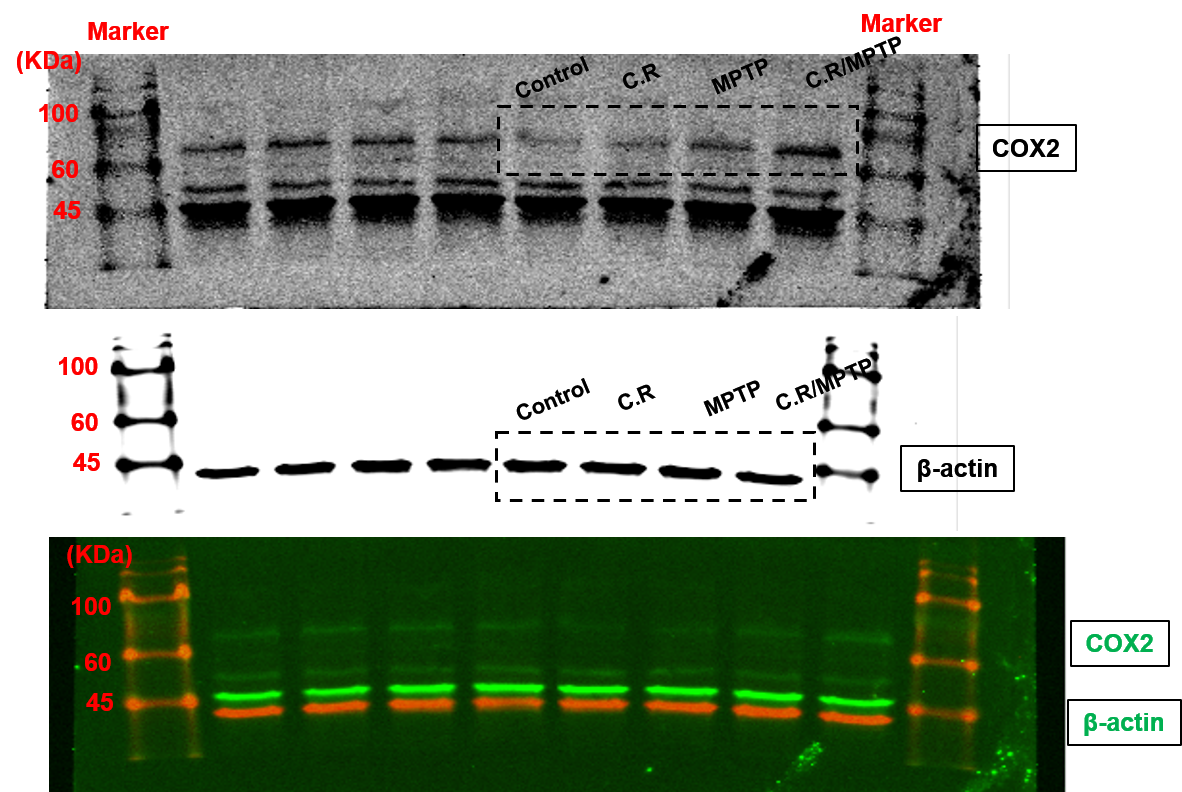
 Fig S16.** The original blot of the **Fig. 10C**.


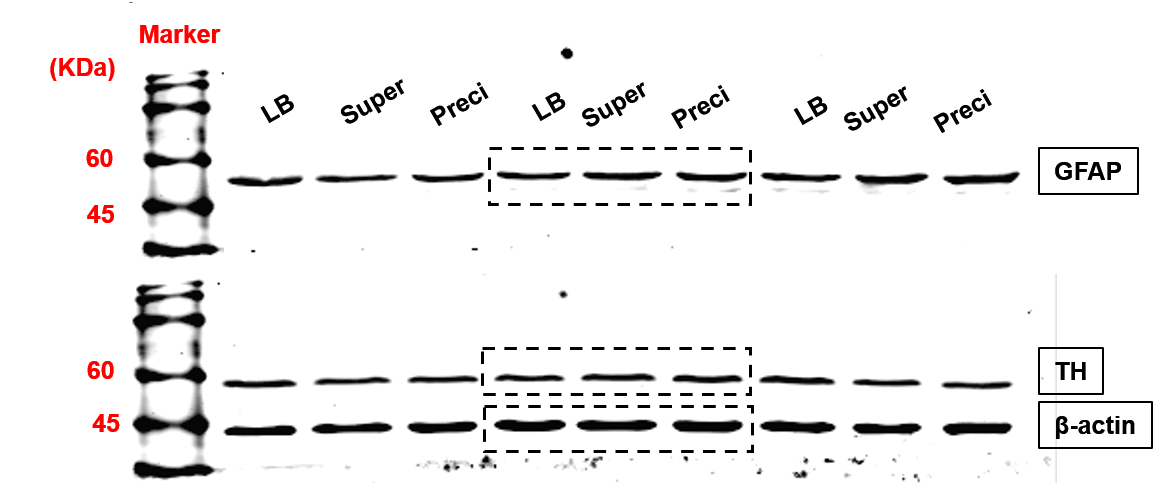
 **Fig S17.** The original blot of the **Fig. S6C**.
